# Quantitative modelling predicts the impact of DNA methylation on RNA polymerase II traffic

**DOI:** 10.1073/pnas.1903549116

**Published:** 2019-07-09

**Authors:** Justyna Cholewa-Waclaw, Ruth Shah, Shaun Webb, Kashyap Chhatbar, Bernard Ramsahoye, Oliver Pusch, Miao Yu, Philip Greulich, Bartlomiej Waclaw, Adrian P. Bird

**Affiliations:** ^a^The Wellcome Centre for Cell Biology, University of Edinburgh, EH9 3BF Edinburgh, United Kingdom;; ^b^Institute of Genetics and Molecular Medicine, University of Edinburgh, Western General Hospital Campus, EH4 2XU Edinburgh, United Kingdom;; ^c^Center for Anatomy and Cell Biology, Medical University of Vienna, 1090 Vienna, Austria;; ^d^Ludwig Institute for Cancer Research, San Diego Branch, La Jolla, CA 92093;; ^e^Mathematical Sciences, University of Southampton, SO17 1BJ Southampton, United Kingdom;; ^f^Institute for Life Sciences, University of Southampton, SO17 1BJ Southampton, United Kingdom;; ^g^School of Physics and Astronomy, University of Edinburgh, EH9 3FD Edinburgh, United Kingdom

**Keywords:** MeCP2, gene regulation, mathematical modelling, DNA methylation

## Abstract

Patterns of gene expression are primarily determined by proteins that locally enhance or repress transcription. While many transcription factors target a restricted number of genes, others appear to modulate transcription levels globally. An example is MeCP2, an abundant methylated-DNA binding protein that is mutated in the neurological disorder Rett syndrome. Despite much research, the molecular mechanism by which MeCP2 regulates gene expression is not fully resolved. Here, we integrate quantitative, multidimensional experimental analysis and mathematical modeling to indicate that MeCP2 is a global transcriptional regulator whose binding to DNA creates “slow sites” in gene bodies. We hypothesize that waves of slowed-down RNA polymerase II formed behind these sites travel backward and indirectly affect initiation, reminiscent of defect-induced shockwaves in nonequilibrium physics transport models. This mechanism differs from conventional gene-regulation mechanisms, which often involve direct modulation of transcription initiation. Our findings point to a genome-wide function of DNA methylation that may account for the reversibility of Rett syndrome in mice. Moreover, our combined theoretical and experimental approach provides a general method for understanding how global gene-expression patterns are choreographed.

## Significance

We introduce an interdisciplinary approach to understanding global modulation of gene expression in mammalian cells. Conventional transcription factors target a limited subset of genes, whereas global modulators bind the genome broadly. An example of the latter is MeCP2, which is mutated in neurological disorders. MeCP2 has millions of genomic binding sites, but its effects on gene expression are mostly small scale and incompletely understood at a mechanistic level. Using datasets from genetically modified human neurons, our mathematical approach rigorously distinguishes global effects from experimental noise. This allows us to integrate theory with experiments to discriminate competing mechanistic models. The results indicate that MeCP2 creates “roadblocks” in gene bodies that slow down elongating RNA polymerase II, leading to polymerase queueing.

Many eukaryotic chromatin-associated factors modulate transcription by binding to specific sites in gene promoters or enhancers (1, 2). Most transcription factors are thought to modulate the initiation rate of transcription by altering histone–DNA interactions (2, 3) or imposing promoter-proximal obstacles (4). However, transcription can also be affected by processes that occur in the bodies of genes. In particular, DNA methylation, which is widespread in gene bodies, appears to affect progression of RNA polymerase II (RNA Pol II) through densely methylated exons (5). The mechanism is unclear, but methyl-CpG binding proteins (6) may be involved. Since most gene bodies contain methylated CpGs, such proteins may have a global effect on transcription.

One putative global modulator is methyl-CpG binding protein 2 (MeCP2) (7, 8), which is highly expressed in neurons. *MECP2* mutations, including loss-of-function or gene duplication, lead to severe neurological disorders (9, 10). MeCP2 does not behave as a conventional transcription factor with discrete targets, as its binding site occurs on average every ∼100 base pairs (bp). Evidence from in vitro systems (11, 12) and mouse models (13, 14) suggests that MeCP2 can mediate DNA-methylation-dependent transcriptional inhibition. Transcriptional changes in mouse brain when MeCP2 is absent or overexpressed are relatively subtle but widespread (15–17), and the molecular mechanisms underlying these changes are unknown.

Here, we set out to resolve the mechanism of MeCP2-dependent transcriptional regulation. Because MeCP2 binding sites occur in the vast majority of genes, we reasoned that most are likely to be influenced to some extent by its presence. To confront the technical and analytical challenges posed by modest changes in the expression of large numbers of genes, we adopted a quantitative approach that combined deep, high-quality datasets obtained from a uniform population of Lund Human Mesencephalic (LUHMES)-derived human dopaminergic neurons (18) with computational modeling. We created a spectrum of LUHMES cell lines expressing distinct levels of MeCP2. Using an assay for transposase-accessible chromatin sequencing (ATAC-seq) and chromatin immunoprecipitation sequencing (ChIP-seq) together with mathematical modeling, we detected a robust footprint of MeCP2 binding to mCG in vivo and determined the amount of MeCP2 bound to DNA. Quantification of mRNA abundance by RNA-sequencing (RNA-seq) revealed a relationship between changes in transcription and the density of mCG on gene bodies. To explain this observation, we proposed and tested several distinct mechanistic models. The only model consistent with our experimental results was one in which MeCP2 leads to slowing down of RNA Pol II progression through a transcription unit. Importantly, mutant MeCP2 that is unable to bind the TBL1/TBLR1 subunits of the NCoR corepressor complex fails to repress efficiently, suggesting that repression depends upon this interaction.

## Results

### Global Changes in Transcription Correlate with MeCP2 Expression Level.

**Fig. 1.**
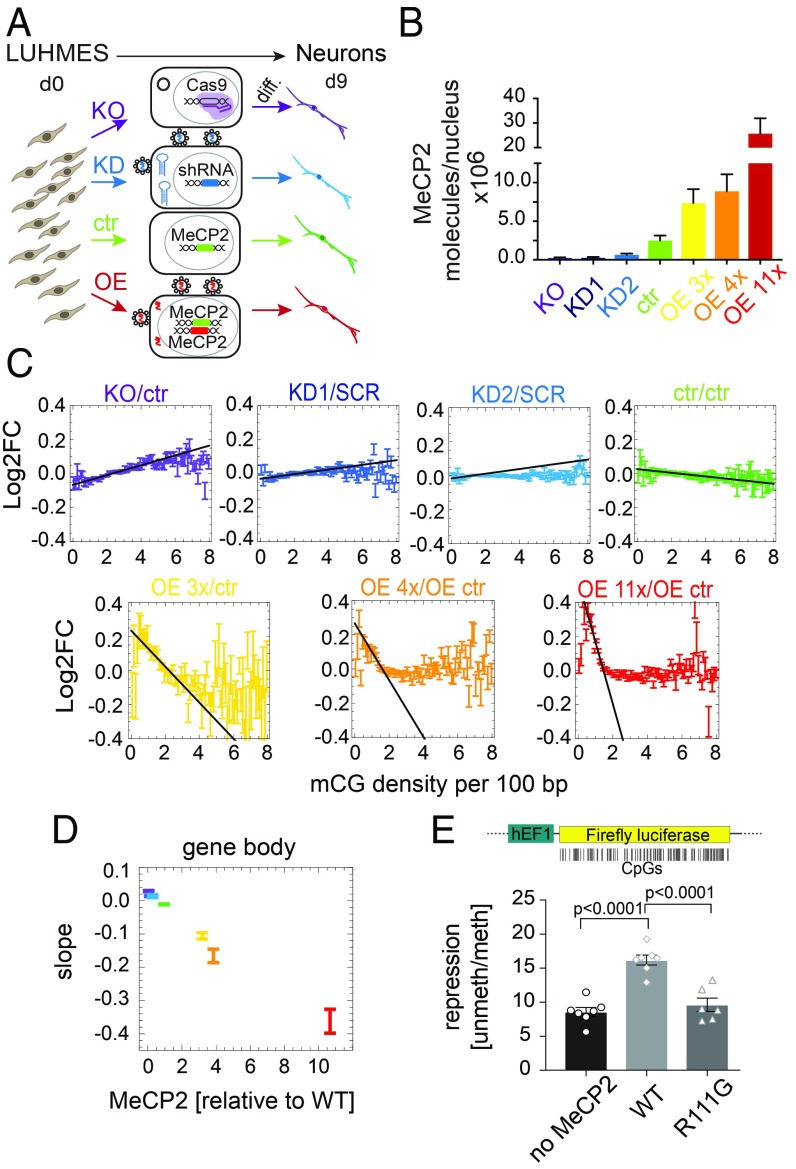
Gene expression strongly correlates with gene body mCG density and MeCP2 abundance. (*A*) Experimental design (*Materials and Methods*). d0, day 0; d9, day 9. (*B*) Mean number of MeCP2 molecules per nucleus. (*C*) Log_2_ fold change of gene expression (Log2FC) relative to appropriate controls (ctr, unmodified controls; SCR, scrambled shRNA control; OE ctr, overexpression control) for all seven levels of MeCP2, plotted against gene body mCG density. All Log2FC values have been shifted so that Log2FC averaged over all genes is zero. Black line indicates the maximum slope. (*D*) The maximum slope for gene bodies varies proportionally to MeCP2 abundance. (*E*) Ratio between luciferase expressions from an unmethylated and gene-body methylated constructs, for three cases: no MeCP2, WT MeCP2, and a methyl-CpG binding domain mutant R111G that is unable to bind mCG. Points show individual replicates. In all panels, error bars represent ±SEM.

We created progenitor cell lines capable of differentiation to a uniform population of human neurons (*SI Appendix*, Fig. S1 *A*–*C*) that expressed seven widely different levels of MeCP2, including knockout (KO), wild-type (WT), and 11-fold overexpression (OE 11x) (Fig. 1 *A* and *B* and *SI Appendix*, Fig. S1*D* and Table S1). All lines differentiated into neurons with similar kinetics, expressed neuronal markers (*SI Appendix*, Fig. S1*E*), and had identical global levels of DNA methylation (∼3.7% of all cytosines were methylated) (*SI Appendix*, Fig. S2*A*). Based on the known affinity of MeCP2 for methylated CG (mCG), we expected that the effect of MeCP2 on gene expression would depend on their mCG content. DNA methylation was therefore quantified for all genes in WT neurons by using whole-genome bisulfite sequencing [Tet-assisted bisulfite sequencing (TAB-seq)] (*SI Appendix*, Fig. S2 *B* and *C*). We calculated total methylation (total mCG, *N*_mCG_) as the number of mCG dinucleotides, mCG density (*ρ*_mCG_) as the number of mCGs per 100 bp, and mCG mean as the percentage of mCG in all CG dinucleotides. To determine the effects of MeCP2 on transcription, we performed RNA-seq on all seven cell lines. We included all expressed protein-coding genes (∼17,000 genes) in our analysis. Most genes responded to MeCP2, but changes were small, precluding definition of a subset of affected genes (*SI Appendix*, Fig. S3*A*). To enhance a possible relationship between expression changes and DNA methylation that otherwise might be obscured by other regulatory mechanisms and statistical noise, genes were binned according to methylation density, considering gene bodies and promoters separately.

The average change in expression vs. appropriate controls [log_2_ fold change of gene expression (Log2FC)] showed a strong relationship to mCG density (*ρ*_mCG_) in gene bodies (Fig. 1*C*). The effect was the strongest for *ρ*_mCG_ = 0.8–4.0 mCG per 100 bp, which includes the vast majority of genes (*SI Appendix*, Fig. S3*B*). The apparent stimulation of expression at very low mCG densities in OE neurons is discussed in *SI Appendix*. Moreover, the maximum slope of the Log2FC vs. *ρ*_mCG_ in gene bodies (Fig. 1 *C*, black lines) was strikingly proportional to MeCP2 levels (Fig. 1*D*). In contrast, plots of Log2FC vs. *ρ*_mCG_ in promoter regions showed a slope close to zero, indicating minimal dependence on promoter methylation (*SI Appendix*, Fig. S3*C*). No clear dependence on MeCP2 level was observed for Log2FC vs. total gene body mCG or mCG mean (*SI Appendix*, Fig. S3 *D* and *E*). These results indicated that the gene-body mCG density is the strongest predictor of MeCP2-dependent transcriptional changes. This relationship was not affected when data were filtered by significance, gene length, or promoter methylation (*SI Appendix*, Fig. S4 *A*–*D*). Moreover, the relationship was maintained even when intronic reads were analyzed, suggesting that pre-mRNA is affected in the same way as processed RNA (*SI Appendix*, Fig. S4*E*). To test for a causal relationship, we transfected cells with two versions (methylated or unmethylated gene body) of a luciferase reporter gene with a methylation-free promoter in the presence of WT or the DNA binding mutant MeCP2 [R111G] (*SI Appendix*, Fig. S5 *A* and *B*). We observed a twofold repression of methylated vs. unmethylated luciferase gene body in the presence of WT MeCP2, compared with either no MeCP2 or mutant MeCP2 (Fig. 1*E*).

### MeCP2 Binds Predominantly mCG Genome-Wide.

**Fig. 2.**
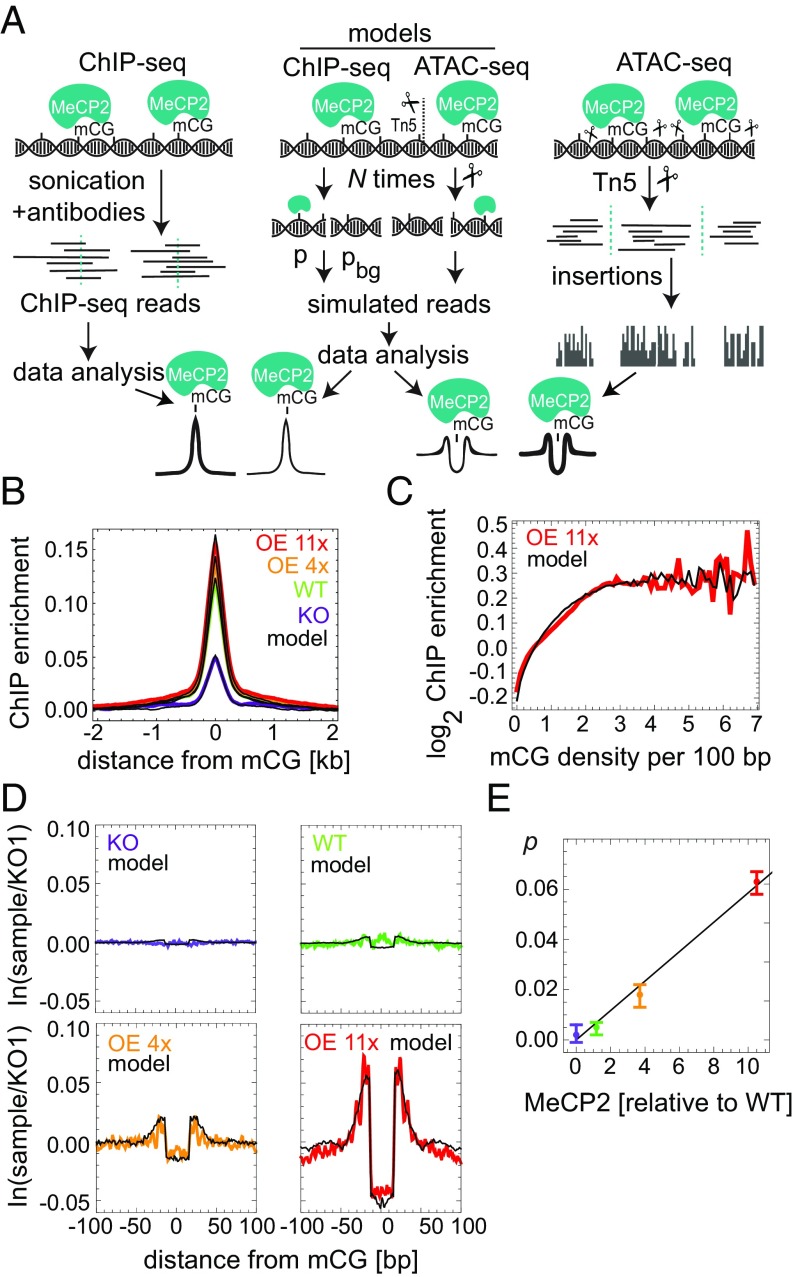
MeCP2 occupancy on the DNA is proportional to mCG density and MeCP2 level. (*A*) MeCP2 ChIP- and ATAC-seq experimental procedures and their in silico counterparts. *p*_*bg*_ and *p* are probabilities of background and MeCP2-bound reads, respectively. Tn5 insertion sites (scissors) occur in exposed DNA regions. (*B*) ChIP-seq enrichment profiles centered at mCG dinucleotides for different cell lines. Black lines represent in silico profiles fitted to the experimental data. (*C*) MeCP2 ChIP-seq enrichment data in OE 11x/KO (red) as a function of mCG density. (*D*) Average depletion profiles (logarithm of the ratio between the number of Tn5 insertions in a given cell line and KO1; two to four biological replicates) in the ±100-bp regions surrounding mCG dinucleotides. Black lines represent computer simulations of the model fitted to the data. (*E*) Predicted fraction of mCGs occupied by MeCP2 vs. MeCP2 level obtained from depletion profiles in *D*. Error bars represent ±SEM.

To map the binding of MeCP2 in human neurons, we performed MeCP2 ChIP-seq for KO, WT, OE 4x, and OE 11x and developed a computer model that simulated the ChIP-seq procedure and MeCP2 binding in vivo (Fig. 2*A*). As expected, ChIP enrichment was proportional to the level of MeCP2 in each cell line (*SI Appendix*, Fig. S6 *A*–*C*) and showed a strong peak centered at the mCGs in MeCP2-positive lines (Fig. 2*B*), as well as a correlation between MeCP2 enrichment and mCG density (Fig. 2*C*). Conversely, enrichment was absent at nonmethylated CGs (*SI Appendix*, Fig. S6*E*).

To derive an independent measure of absolute MeCP2 density on the DNA and to detect its molecular footprint with high resolution, we performed ATAC-seq (19), in which transposase Tn5 cuts exposed DNA to reveal DNA accessibility within chromatin (Fig. 2*A*). In agreement with the ChIP-seq data, ATAC-seq Tn5 insertion profiles (Fig. 2*D*) showed a graded depletion of insertion sites centered around mCG in WT, OE 4x, and OE 11x neurons, whose amplitude was proportional to MeCP2 concentration (Fig. 2*E*) and therefore represented a “molecular footprint” of MeCP2 binding in vivo. The size and amplitude of the footprint agreed well with a computer model of ATAC-seq and MeCP2 binding (Fig. 2 *D*, black lines) and previous in vitro data (20, 21), confirming that MeCP2 occupies 11 bp of DNA in living cells. No depletion of insertion sites was observed over unmethylated CG (*SI Appendix*, Fig. S6*F*). The model revealed that only 6.3% of mCG sites are actually occupied by MeCP2 in OE 11x neurons, falling to <1% occupancy in WT (Fig. 2*E*), perhaps due in part to occlusion by nucleosomes. Excellent agreement between the models and ATAC-seq and ChIP-seq data allowed us to predict MeCP2 occupancy from mCG density and MeCP2 level in each cell line (Fig. 2*E* and *SI Appendix*, Fig. S6*D*).

### MeCP2 Does Not Regulate Transcription via Condensation of Chromatin or Premature Termination.

**Fig. 3.**
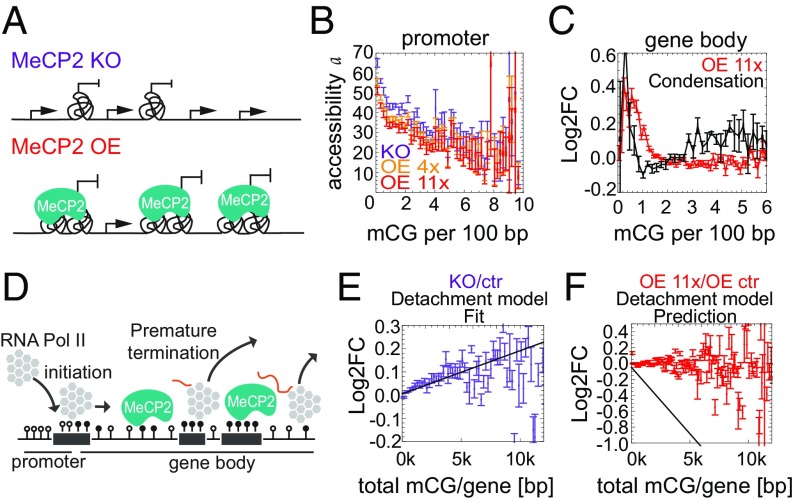
MeCP2 does not regulate transcription via condensation of chromatin or premature termination. (*A*) A cartoon of the condensation model. Tangles represent regions of condensed chromatin that are inaccessible to RNA Pol II. (*B*) Chromatin accessibility (measured by ATAC-seq) at promoters rapidly decreases with increasing promoter methylation. In contrast, MeCP2 has a minor effect on accessibility (curves for OEs 4x and 11x are slightly lower than for KO). (*C*) The condensation model disagrees with Log2FC(OE 11x/KO) obtained from RNA-seq. (*D*) Schematic representation of the detachment model. (*E*) Log2FC (gene expression) for KO/ctr (purple) vs. the total number of mCGs per gene. Black lines represent predictions of the detachment model. Error bars represent ±SEM. (*F*) As in *E* for OE 11x/OE ctr (red).

To interpret these results mechanistically, we considered mathematical models based on a commonly accepted paradigm for gene expression (*SI Appendix*, Fig. S7*A*) (22). In the first class of models, named condensation models (Fig. 3*A*), MeCP2 affects the rate of transcription initiation via changes in chromatin structure. The possibility that MeCP2 affects the initiation rate *α* by binding to promoters was rejected because it would imply a stronger correlation between gene expression and *ρ*_mCG_ in promoters than in gene bodies, contrary to our observations (*SI Appendix*, Fig. S3*C*). MeCP2 could hypothetically affect the fraction *f* of cells with specific genes in the ON state via some long-distance mechanism involving binding to gene bodies and leading to changes in the degree of chromatin openness near promoters. However, mapping chromatin accessibility by using ATAC-seq showed that, while there is a weak correlation between MeCP2 and accessibility (Fig. 3*B*), it cannot account for the observed Log2FC in gene expression (Fig. 3*C*).

We next considered potential effects of MeCP2 on the elongation phase of transcription. The detachment model posits that MeCP2 causes transcription to prematurely abort (Fig. 3*D*). Since the probability of termination increases with each blocking site, under this model, the Log2FC is a function of the total number of mCGs (*N*_mCG_) in the gene: Log2FC = −*γ* (*M* − 1)*N*_mCG_, where *M* is MeCP2 concentration relative to WT, and the parameter *γ* is proportional to the probability that Pol II aborts transcription when it encounters MeCP2 or an MeCP2-induced chromatin modification. The unknown parameter *γ* can be obtained by fitting the model to the Log2FC (KO/WT) data (Fig. 3*E*). We found that the model failed to reproduce the Log2FC vs. *N*_mCG_ relationship for the OE 11x cell line (Fig. 3*F*). The model also failed to correctly predict the observed relationship between Log2FC and mCG density in gene bodies (*SI Appendix*, Fig. S7 *B* and *C*). Therefore, it is unlikely that MeCP2 affects transcription via premature termination.

### MeCP2 Creates “Dynamical Obstacles” That Impede Transcriptional Elongation.

**Fig. 4.**
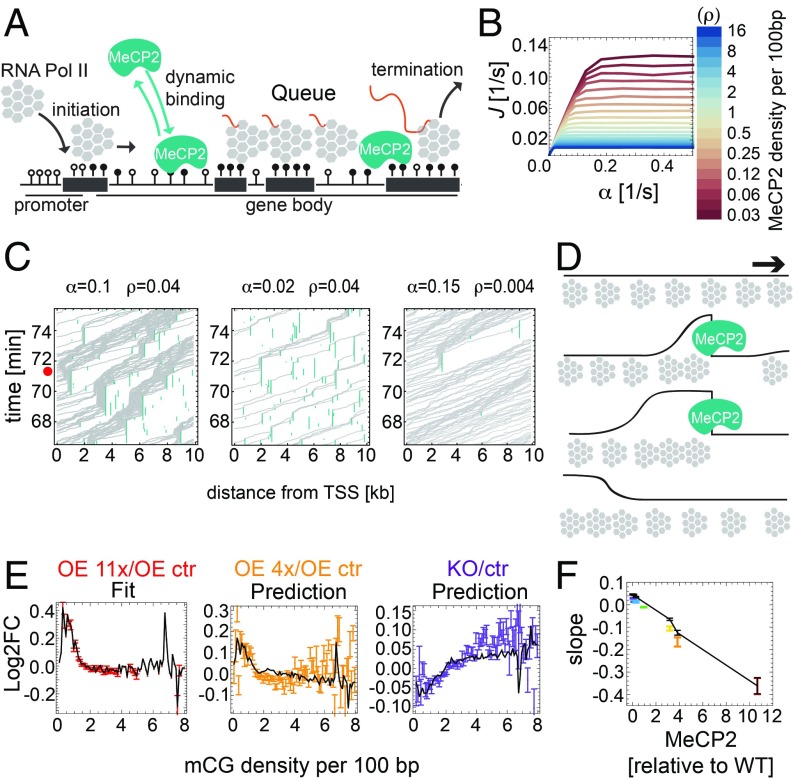
Mathematical modeling indicates that MeCP2 slows down transcriptional elongation. (*A*) Schematic representation of the dynamical obstacles model. (*B*) Transcription rate *J* predicted by the model, plotted as a function of the initiation rate *α*, for different mean MeCP2 densities in gene bodies. (*C*) Space–time plots (kymographs) representing Pol II moving along the gene. Queues of Pol II induced by MeCP2 can reach the TSS (red dot) and block initiation if both the initiation rate (α) and the density of MeCP2 (ρ) are sufficiently high (*C*, *Left*). (*D*) Schematic representation of Pol II (gray) density shockwaves forming behind MeCP2 (blue). Black line is the local density of Pol II. (*E*) Log2FC (gene expression) vs. mCG density in gene bodies obtained in computer simulations of the dynamical obstacles model (black solid lines) fitted to the OE 11x/OE ctr RNA-seq dataset (red) agrees well with experimental data for OE 4x/OE ctr (orange) and KO/ctr (purple) datasets. Error bars represent ±SEM. (*F*) The maximum slope of Log2FC (gene expression) vs. mCG density in gene bodies, predicted by the dynamical obstacles model (black line). Points are experimental slopes from Fig. 1*C*.

Finally, we considered a “congestion model,” whereby Pol II pauses when it encounters MeCP2 itself or an induced, transient structural modification of chromatin (Fig. 4*A*). The parameters were: the fraction *p* of mCGs bound by MeCP2, MeCP2 turnover (unbinding) rate *k*_*u*_, and (specific to each gene) the length *L* of the gene, the density *ρ*_mCG_ of mCGs, and the initiation rate *α*. Fig. 4*B* shows the transcription rate for OE 11x predicted by the model as a function of *α*, for different mean MeCP2 densities (*pρ*_mCG_). The assumed value of *k*_*u*_ = 0.04 s^−1^ is compatible with the reported in vivo residence time of MeCP2 on chromatin [25–40 s (23)]. Inspired by nonequilibrium statistical mechanics approaches that have been utilized to model one-dimensional transport (24, 25), we expected a nonequilibrium phase transition from a low-density to a maximal-current (congested) phase as the initiation rate or the density of obstacles increase beyond a critical point. Indeed, all curves in Fig. 4*B* have a characteristic shape: a linear relationship *J* ∼ *α* for small *α*, followed by saturation at high initiation rates. Saturation occurs due to congestion as polymerases queue upstream of obstacles (Movies S1 and S2). However, even in the nonsaturated regime of intermediate *α*, excluded-volume interactions between polymerases that have been slowed down by an obstacle cause a density shockwave that propagates backward (Fig. 4*C*). A small increase in the density of polymerases near the promoter decreased the rate of Pol II binding to the transcription start site (TSS). Thus, even though MeCP2 does not directly affect Pol II initiation, it does so indirectly by shockwaves that form behind MeCP2-induced obstacles in gene bodies (Fig. 4*D*). To test the model against RNA-seq data, we estimated average initiation rates for genes with similar mCG densities by fitting the model to Log2FC data from one of the cell lines [OE 11x/OE control (ctr); Fig. 4 *E*, *Left* and *SI Appendix*, Fig. S8*F*]. We then used the model to predict Log2FC for the remaining six cell lines. The model strikingly reproduced the data (Fig. 4*E* for OE 4x and KO) as well as the slopes of the Log2FC plots for all seven cell lines (Fig. 4*F*). A similar behavior occurred in a modified model in which Pol II slowed down (rather than completely stopped) on permanent or long-lasting structural modifications of chromatin (*SI Appendix*, Fig. S8 *A*–*E* and Movie S3). We conclude that both congestion models are compatible with the experimental data presented in Fig. 1 *C* and *D*. The models also predict that Log2FC should decrease with increasing expression (measured as transcripts per million reads), in agreement with the data (*SI Appendix*, Fig. S8*G*).

### MeCP2 Binding to Both DNA and NCoR Are Essential to Slow Down RNA Pol II.

**Fig. 5.**
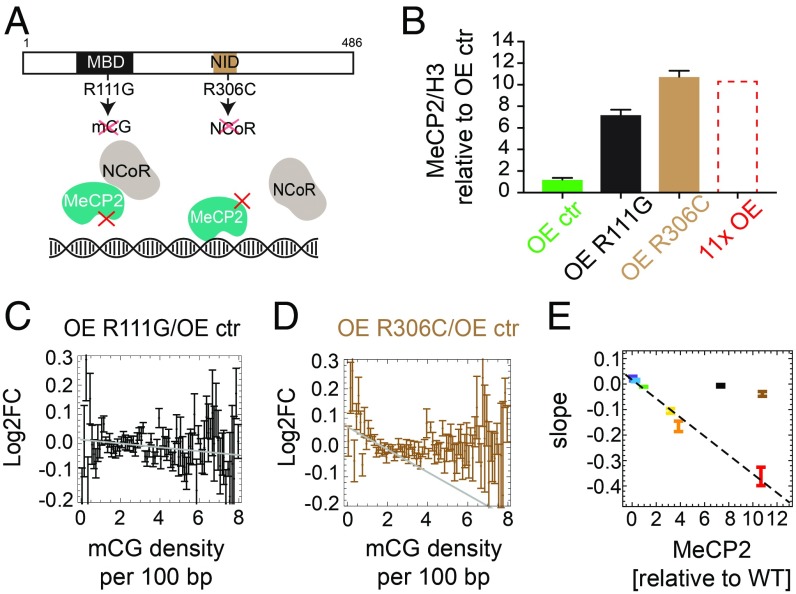
MeCP2 slows down transcription via a mechanism involving NCoR. (*A*) Location of two binding domains in MeCP2 that are relevant for the proposed mechanism: methyl-CpG binding domain (MBD) and NCoR-interaction domain (NID). The mutation R111G causes MeCP2 to lose the ability to bind specifically to mCG. The mutation R306C prevents MeCP2 from binding the NCoR complex. (*B*) Level of MeCP2 (Western blot) in two overexpressed mutant cell lines (R111G and R306C) and the overexpression control cell line (OE ctr). OE 11x is shown for comparison. Values are averaged over three biological replicates and normalized by the level of histone H3. (*C*) Log2FC (expression) of OE R111G/OE ctr shows almost no dependence on mCG density in gene bodies (black). The gray line shows the maximum slope. (*D*) Log2FC (expression) of OE R306C/OE ctr shows a small negative correlation with gene body mCG density (brown). The gray line shows the maximum slope. (*E*) Maximum slopes for all cell lines including OE R111G (black) and OE R306C (brown) from *C* and *D* vs. MeCP2 level (Western blot). In all plots, error bars represent ±SEM.

To address the question of whether MeCP2 impedes Pol II progression directly by steric interference or indirectly by altering chromatin structure [e.g., by histone deacetylation (26)], we overexpressed mutated forms of MeCP2 in the presence of WT MeCP2. The mutants were either unable to bind methylated DNA (R111G) (27) or unable to recruit the histone deacetylase complex NCoR (R306C) (14, 28) (Fig. 5*A* and *SI Appendix*, Fig. S9*A*). As expected, sevenfold overexpression of MeCP2–R111G caused no mCG-density-dependent transcriptional changes (Fig. 5 *B* and *C* and *SI Appendix*, Fig. S9 *B* and *C*). The R306C mutant, on the other hand, was predicted to repress transcription if inhibition is directly due to MeCP2 binding to DNA, but not if inhibition is mediated via the corepressor. In fact, 11-fold overexpression of MeCP2–R306C relative to WT MeCP2 caused only a small perturbation of gene expression, indicating a significant loss of DNA methylation-dependent repression (Fig. 5 *B* and *D* and *SI Appendix*, Fig. S9 *B* and *C*). The weak slope may represent minor direct interference of DNA-bound MeCP2–R306C with transcription. As neither mutant falls on the line defining the linear relationship between gene repression and MeCP2 concentration (Fig. 5*E*), our findings favor a predominantly indirect mechanism of repression, whereby corepressor recruitment alters the chromatin state to impede transcription.

## Concluding Remarks

In summary, a close alliance between mathematical modeling and molecular biology has allowed us to discriminate molecular mechanisms underlying the relatively subtle global effects of MeCP2 on global gene expression. The proposed mechanism relies on MeCP2–NCoR interaction that slows down the progression of Pol II during transcription elongation. A candidate mediator of this effect is histone modification, in particular histone deacetylation, as cell-transfection assays using methylated reporters demonstrate that repression depends upon histone deacetylase activity (11, 12). According to this scenario, MeCP2 recruitment of the histone deacetylase corepressor NCoR would restrain transcription, perhaps by causing tighter binding of nucleosomes to DNA (26). To explain the dramatic reversibility of Rett syndrome in animal models (29), we propose that, in the absence of MeCP2, DNA methylation patterns are unaffected, allowing the reexpressed WT protein to bind within gene bodies and commence normal modulation of transcriptional elongation. We suggest that the congestion model may apply to proteins other than MeCP2. For example, other chromatin-binding factors that bind short (and thus abundant) motifs, including other methyl-binding proteins, may modulate gene expression by a similar mechanism.

## Materials and Methods

### Cell Lines.

The procedure for culture and differentiation of the LUHMES cell line was described (18). To create two independent *MECP2* KO lines, we used CRISPR-mediated gene disruption (30). To generate MeCP2 knockdowns, several shRNAs against MeCP2 were designed by using Sigma-Aldrich Mission shRNA online software. Two shRNAs were chosen and cloned into a pLKO.1 vector including scrambled shRNA as a control, and lentiviruses were created (*SI Appendix*, Table S2). To increase the level of MeCP2, we created lentiviruses expressing MeCP2 from two alternative promoters in the pLKO.1 vector: synapsin and cytomegalovirus. Calculation of SD, SEM, and *t* tests for qPCR, Western blots, methylation, and total RNA quantification using high-performance liquid chromatography (HPLC) were performed by using GraphPad Prism (version 7).

### Repression Assay.

CpG-free vector containing Firefly Luciferase with CpGs was methylated by M.SssI methyltransferase in the presence or absence of *S*-adenosyl-l-methionine. Mouse embryonic fibroblasts were transfected by using Lipofectamine 2000 with three plasmids containing Firefly Luciferase, Renilla Luciferase, and MeCP2. Luciferase activity measurements were performed by using the Dual Luciferase assay kit (Promega), according to manufacturer’s protocol.

### Library Preparation for Illumina Sequencing.

All libraries were sequenced as 75- or 100-nucleotide-long paired-end reads on HiSeq 2000 and HiSeq 2500 Illumina platforms. Methylome of WT LUHMES-derived neurons at day 9 was obtained by TAB-seq according to the published protocol (31). The RNA-seq library was prepared according to manufacturer’s protocol for the ScriptSeq Complete Gold kit (human/mouse/rat). Total RNA was isolated from all generated cell lines (*SI Appendix*, Table S1) at day 9 of differentiation by using either the RNeasy Mini kit or the AllPrep DNA/RNA Mini kit (Qiagen). ATAC-seq in four cell lines (KO, WT, OE 4x, and OE 11x; *SI Appendix*, Table S1) was performed as in ref. 32.

MeCP2 ChIP-seq was performed by using LUHMES-derived neurons at day 9 of differentiation with four levels of MeCP2: KO, WT, OE 4x, and OE 11x (*SI Appendix*, Table S1). Libraries were prepared by using the NEBNext Ultra II DNA library Prep kit (NEB) for both immunoprecipitations and corresponding inputs.

### Data Processing of Raw Reads from Illumina Sequencing.

All reads were quality-controlled, trimmed to remove adapters (Trimmomatic) (33) and duplicated reads, and mapped to the human hg19 reference genome. Bismark (34) was used to extract cytosine methylation from TAB-seq. All raw data were deposited in the Gene Expression Omnibus database (accession no. GSE125660) (35).

### RNA-Seq Data Analysis.

We used a subset of protein-coding genes with sufficient methylation coverage (bisulfite sequencing; ≥80% C with coverage ≥20), and gene bodies 1 kb or longer. This resulted in 15,382 genes of the initial 17,764 protein-coding genes (86%). In all plots of Log2FC of differential gene expression, we shifted the Log2FC values so that the average Log2FC in the range of mCG density *ρ*_mCG_ ∈ [1,6]/100 bp was zero for all samples. This was motivated by the difficulty in determining the absolute levels of expression, since we did not quantify total mRNA.

### ChIP-Seq Enrichment Profiles.

We first obtained accumulated counts (the number of reads) $c_{i}^{x}$ that overlapped with *i*-th base pair to the right (*i* > 0) or left (*i* < 0) from feature *x*(*x* = mCG, non-mCG,...). We then calculated enrichment profiles as

$$f_{i}=\frac{{\text{Norm}}_{1}\left(c^{\text{ChIP},x}\right)\left[i\right]}{{\text{Norm}}_{1}\left(c^{\text{input},x}\right)\left[i\right]}-1,$$

where $c_{i}^{\text{ChIP},x}$ and $c_{i}^{\text{input},x}$ are accumulated counts from ChIP and input (genomic) DNA sequencing, respectively, and Norm_1_(*c*)[*i*] normalizes the counts profiles such that their flanks have values close to one:

$${\text{Norm}}_{1}\left(c\right)\left[i\right]=\frac{c_{i}}{\left(\sum_{j=-500}^{-301}c_{j}+\sum_{j=301}^{500}c_{j}\right)/400}.$$

We considered a particular C to be methylated if it was methylated in 100% of the reads, and the coverage was at least 5. We considered a C to be unmethylated if it did not show up in any of the ChIP-seq reads as methylated.

### Computer Model of ChIP-Seq.

We assumed that MeCP2 occupies methylated cytosines with probability *p* times the probability of binding to a particular motif. Binding probabilities for different motifs are based on known binding affinities (36) and relative binding strengths (15). To create simulated ChIP fragments, we assumed that if a DNA fragment contained at least one MeCP2 bound to it, it would be present in the simulated ChIP-seq. Fragments that do not contain any MeCP2 may still be present in the ChIP-seq data with probability *p*_*bg*_, which accounts for “background” reads in ChIP-seq, even in the absence of MeCP2. This is similar to previous models of ChIP-seq (37); even the best ChIP-seq libraries can have a significant level of background reads (*p*_*bg*_ close to 1) (38). We also added CG and length bias and processed simulated reads in the same way as the experimental ChIP data.

For each ChIP-seq dataset, we fitted the simulated profile (parametrized by *p*, *p*_*bg*_) to the experimental profile. Any *p* ≤ 0.1 gives a good fit (*SI Appendix*, Fig. S6*D*), indicating that *p* ∼ 0.1 is the upper bound on mCG occupancy in 11x OE. We used best-fit parameters to predict profiles on features other than mCG (*SI Appendix*, Fig. S6*E*).

### ATAC-Seq Footprints.

ATAC-seq was analyzed in a similar way to ChIP-seq, except that we used fragments’ endpoints (Tn5 insertion sites) to generate accumulated counts *n*_*i*_. We calculated the insertion profiles as

$$f_{i}=\ln\left[\frac{{\text{Norm}}_{2}\left(n_{i}^{\text{cell line}}\right)}{{\text{Norm}}_{2}\left(n_{i}^{\text{KO}}\right)}\right],$$

where $n_{i}^{\text{cell line}}$ and $n_{i}^{\text{KO}}$ are the insertion counts profiles for a given cell line and KO1, respectively, and Norm_2_ normalizes the counts profiles such that their flanks have values close to one:

$${\text{Norm}}_{2}\left(n_{i}\right)=\frac{n_{i}}{\left(\sum_{j=-50}^{-41}n_{j}+\sum_{j=41}^{50}n_{j}\right)/20}.$$

### Computer Model of ATAC-Seq.

We used the same binding model as in the ChIP-seq simulations. We assumed that MeCP2 occupies 11 bp (20) and that the protein is centered on an mC. We simulated the action of the Tn5 transposase by splitting the sequence into fragments in areas free of MeCP2, and we included Tn5 sequence bias and CG and length bias. The model has three parameters: the density *p* of MeCP2 on mCxx, the average density of insertion (cut) sites *t*, and the GC bias *b*. We processed simulated DNA fragments in the same way as described above for the experimental data. We examined the role of the parameters on the shape and depth of the simulated footprint of MeCP2 and concluded that the footprint is not affected as long as the test and control samples have been processed in a similar way. To extract MeCP2 occupancy *p* from ATAC-seq data, we fitted the model (free parameters *p*, *t*, and a fixed *b* = 6.0) to experimental footprints for all four cell lines. The relationship is linear (Fig. 2*E*), with the best-fit *p* = 0.0058 × *M*_cell_ _line_/*M*_WT_.

### Chromatin Accessibility from ATAC-Seq.

For each gene, we calculated its mean insertion count $\bar{n}$ and selected regions (“insertion peaks”) in which $n_{i}>4\bar{n}$. Accessibility was defined as the sum of all insertions in the peaks divided by the “background” $\bar{n}$:

$$a=\frac{\sum_{i}n_{i}}{\bar{n}}.$$

### Mathematical Models of Gene Expression.

The condensation model assumes that the fraction *f*_*i*_ of cells in which gene *i* is actively transcribed depends on promoter openness *a*_*i*_ (measured by ATAC-seq), which in turn depends on the level *M* of MeCP2 and gene methylation *ρ*_*i*_: *f*_*i*_ = *f*_*i*_(*M*, *ρ*_*i*_) ∝ *a*_*i*_ = *a*_*i*_(*M*, *ρ*_*i*_). The model predicts that Log2FC_X/Y_ of the ratio of gene expression of cell line X vs. cell line Y should yield the same curve (plus a constant) as the logarithm of the ratio of accessibilities of X vs. Y when plotted as a function of *ρ*_mCG_. Data did not support this model (Fig. 3*C*). The detachment model poses that the probability that RNA Pol II successfully terminates is $P={\left(1-\lambda \right)}^{n}≅e^{-\lambda n},$ where *n* is the number of “abort sites” on the gene, proportional to the number of MeCP2 molecules on the gene, and *λ* is the abortion probability. We show that

$$\text{Log}2{\text{FC}}_{\text{X}/\text{Y}}=\text{const}-\gamma \left(\frac{M_{\text{X}}}{M_{\text{Y}}}-1\right)n,$$

where *γ* ∝ *λ* is an unknown parameter identical for all cell lines, and *M*_X_, *M*_Y_ are MeCP2 levels in cell lines X and Y. The model was rejected (Fig. 3*F*).

We considered two mechanisms by which MeCP2 could affect elongation. To implement the slow-sites model, we used the totally asymmetric simple exclusion process (TASEP) with open boundaries (24). A gene is represented as a chain of *L* sites. Each site (equivalent to 60 bp of the DNA) is either occupied by a particle (RNA Pol II) or is empty. Particles enter the chain at site *i* = 1 with rate *α* (transcription initiation rate), move along the chain, and exit at site *i* = *L* with rate *β* = 1 s^−1^. Sites can be “fast” or “slow.” Slow sites represent mCGs affected by the interaction with MeCP2, whereas fast sites are all other sites (methylated or not). Particles jump with rate *v* = 1 s^−1^ (equivalent to Pol II speed ∼60 bp/s) on fast sites and *v*_*s*_ = 0.05 s^−1^ on slow sites. Slow sites are randomly and uniformly distributed with density *ρ*_*s*_ = *pρ*_mCG_, where *p* is the probability that an mCG is occupied by MeCP2. To relate this model to the mRNA-seq differential expression data, we calculated Log2FC as

$$\text{Log}2{\text{FC}}_{\text{X}/\text{Y}}=\log_{2}\frac{J\left(\alpha ,\rho_{\text{s},\text{X}}\right)}{J\left(\alpha ,\rho_{\text{s},\text{Y}}\right)},$$

where *ρ*_s,X_ = *ρ*_mCG_*p*_X_, *ρ*_s,Y_ = *ρ*_mCG_*p*_Y_, in which *p*_X_, *p*_Y_ are MeCP2 occupation probabilities for cell lines X, Y. In the above expression, we know all quantities except the initiation rate *α*, which we fit to the OE 11x data.

The dynamical obstacles model is very similar, with two exceptions: (*i*) Pol II always moves with the same speed *v* (no slow sites) as long as it is not blocked by other polymerases and obstacles; and (*ii*) obstacles bind and unbind dynamically from the methylated sites. We assumed that unbinding occurs with rate *k*_*u*_ per obstacle, whereas binding occurs with rate *k*_*u*_*p* per unoccupied mCG. Obstacles do not bind if an mCG is already occupied by an obstacle or a polymerase. We assumed that obstacles are not restricted to accessible mCGs and that their density on actively transcribed genes may be higher than *p* obtained from ATAC-seq, but still proportional to MeCP2 level. We found that *p* = *M*/*M*_OE11x_ reproduces Log2FC data for all cell lines. Computer programs, scripts, and data related to mathematical models have been deposited at the Edinburgh Data Share database (39).

Additional details for materials and methods are provided in *SI Appendix*.

## Supplementary Material

Supplementary File

Supplementary File

Supplementary File

Supplementary File
