# Supplementary material for: Quantitative modelling predicts the impact of DNA methylation on RNA polymerase II traffic
Source: Proc Natl Acad Sci U S A. 2019 Jul 9;116(30):14995–5000. doi: 10.1073/pnas.1903549116 (PMC6660794; doi:10.1073/pnas.1903549116)
Supplement: Supplementary File [file pnas.1903549116.sapp.pdf]

PNAS
www.pnas.org

Supplementary Information for

Quantitative modelling predicts the impact of DNA methylation on RNA polymerase II traffic

Justyna Cholewa-Waclaw, Ruth Shah, Shaun Webb, Kashyap Chhatbar, Bernard Ramsahoye, Oliver Pusch, Miao Yu, Philip Greulich, Bartlomiej Waclaw, and Adrian P. Bird

Bartlomiej Waclaw

Email: bwaclaw@ph.ed.ac.uk

Adrian Bird

Email: a.bird@ed.ac.uk

This PDF file includes:

Supplementary text

Figs. S1 to S13

Tables S1 to S4

Captions for movies S1 to S3

References for SI reference citations

Other supplementary materials for this manuscript include the following:

Movies S1 to S3

Supplementary Information Text

1. Cell lines and experimental methods.

1.1. LUHMES culture and differentiation.

Proliferating LUHMES cells were cultured on Nunc plasticware coated with 40 µg/ml Poly-L-ornithine (Sigma-Aldrich; P-3655-100mg) and 1µg/ml Fibronectin (Sigma-Aldrich; F-1141-5mg) in medium for proliferating cells: Advanced DMEM (Life Technologies; 12634-010) plus N2 supplement (Life Technologies; 17502-048), 2mM L-Glutamine (Sigma-Aldrich; G7513), and 40 µg/ml bFGF (R&D Systems; 4114-TC-1 mg). For differentiation, LUHMES cells were seeded on 40 µg/ml Poly-L-Ornithine and 1 µg/ml Fibronectin coated plastic-ware at a density of 3-4 x 10^4 cells/cm^2 in medium for proliferating cells. Next day after seeding, the medium was changed to the differentiation medium containing Advanced DMEM, 2mM L-Glutamine, N2 supplement, 1mM cAMP (Sigma-Aldrich; D0627), 1µg/ml Tetracycline (Sigma-Aldrich; T-7660) and 2 ng/ml GDNF (R&D Systems; 212-GD-50 µg). Two days later, cells were trypsinized and seeded at a density of 1.1x10^5 cells/cm^2 in the differentiation medium for the final differentiation (Fig. S1A).

1.2. Lentiviral particle preparation and LUHMES infection.

Lentiviruses were produced in HEK293FT cells (Life Technologies; R700-07) by retrograde transfection of vectors: 7.5 µg pLKO.1; 4.6 µg psPax2; 2.8 µg pMD2G using Lipofectamine 2000 (Life Technologies; 11668027). For one transfection, plasmids in appropriate ratios were added to 1.5 ml OptiMEM (Life Technologies; 31985062) and 36 µl of Lipofectamine 2000 was mixed with 1.5 ml of OptiMEM. Both solutions were combined and incubated for 20 min at room temperature. Meanwhile, HEK293FT cells were trypsinised and concentration-adjusted to 1.2x10^6/ml. 5 ml of cell suspension was added to a 10 cm dish; the transfection mix was then added and the culture was left overnight. Cells were left for 72 hours, after which the supernatant was collected, and viral particles were precipitated using a PEG Virus Precipitation kit (BioVision) following the manufacturer’s protocol. Viruses were aliquoted and either used directly or frozen at -80 ºC. LUHMES cells were infected with lentiviral particles overnight. The next day, cells were selected with 0.5 µg/ml Puromycin (Life Technologies; A11138-03), and concentration of antibiotic was reduced the following day to 0.25 µg/ml. The population of resistant cells was either analysed as a pool or FACS-sorted into a 96-well plate in order to isolate single clones.

1.3. Western blot, qPCR, immunofluorescence, live cell imaging.

For protein quantification, cells were either lysed to give a whole cell extract or only nuclei were isolated, depending on the location protein of interest. To obtain whole cell extracts, cells were homogenized in the NE1 buffer (20 mM HEPES pH 7.9, 10 mM KCl, 1 mM $MgCl_2$, 0.5 mM DTT, 0.1% Triton X, 20% glycerol, 2mM PMSF, protease inhibitor) and treated with Benzonase (Sigma) for 15 min at RT to remove DNA. Protein concentration was measured (Bradford, Bio-Rad) and loading buffer (5x, 250 mM Tris·HCl pH 6.8, 10% SDS, 30% [v/v] Glycerol, 10 mM DTT, 0.05% [w/v] Bromophenol Blue) was added. Samples were boiled for 5 min prior to loading 40 µg of protein extract onto a polyacrylamide gel.

To prepare nuclei, cells were lysed in hypotonic buffer (0.32 M Sucrose, 10 mM Tris-HCl pH 8, 3 mM $CaCl_2$, 2 mM MgOAc, 0.1 mM EDTA, 0.5% NP-40). Nuclei were washed with NP-40-free buffer, counted, centrifuged, and resuspended in an appropriate volume of 20% glycerol in PBS. Nuclei were treated with Benzonase, loading buffer was added and samples were boiled for 5 min. Nuclear lysates ($1.6x10^5$ per lane) were separated on the gradient 4-15% SDS-PAGE gels (Mini-Protean, Bio-Rad) in running buffer (25 mM Tris, 190 mM glycine, 0.1% SDS) at 200 V for ~40 min alongside a protein size marker (PageRuler, Thermo Scientific). Proteins were transferred onto a Nitrocellulose membrane using wet transfer method for 1 hour at 200 V. The membrane was stained first with Ponceau S solution for quality check and then treated with blocking buffer (PBS, 1% PVP, 1% non-fat dried milk, 0.1% Tween 20, 0.01% NaN_3) for 30 min at RT. The membrane was incubated for 1 hour with primary antibodies (see Table S3) at room temperature and then 1 hour with secondary antibodies conjugated with either IRDye 700DX or IRDye 800CW (LI-COR). The membrane was washed with PBS containing 0.1% Tween 20 after adding each antibody and was imaged using Odyssey CLx (LI-COR).

For qPCR analysis, total RNA was isolated using RNeasy kit (Qiagen) and treated with DNase I (DNA free kit, Ambion) to remove genomic DNA. The efficiency of removal of genomic DNA in the samples was tested by performing PCR using primers against the *GAPDH* genomic locus. Synthesis of cDNA was performed using qScript cDNA Supermix (Quanta Biosciences) from 1 µg of total RNA according to the manufacturer's protocol. cDNA was diluted 100x and 2.5 µl aliquots subjected to qPCR with SensiMix SYBR & Fluorescein Mastermix (Bioline) and appropriate primers (see Table S4) on a LightCycler 480 (Roche).

For immunofluorescence analysis, cells were seeded on coverslips and either fixed the next day for undifferentiated LUHMES cells or differentiated for defined time points and then fixed with 4% formaldehyde for 10 min at room temperature. Cells were permeabilised with 0.2% Triton X in PBS for 10 min and blocked with 10% fetal bovine serum in PBS for 30 min at RT.

Primary antibody (Table S3) incubation was performed in 1% FBS, 0.1% Tween 20 in PBS for 1 hour at RT and coverslips were washed three times with 0.1% Tween 20 in PBS. Secondary antibodies (Alexa Fluor 488, Alexa Fluor 555, Alexa Fluor 647, Thermo Scientific) were diluted 1000x in 1% FBS, 0.1% Tween 20 in PBS and applied for 1 hour at RT. Finally, cells were washed three times with 0.1% Tween 20 in PBS, stained with 5000-fold diluted DAPI in PBS for 10 min at RT, mounted using Prolong Gold or Diamond (Thermo Scientific) and dried overnight at RT. Cells were imaged on the Leica TCS Sp5 confocal microscope (Leica Microsystems) using either 40x or 63x oil immersion objectives. Image analysis was performed using Volocity (PerkinElmer). Live cell imaging of differentiating neurons was performed using the IncuCyte Zoom system (Essen BioScience) and neurite lengths were analysed using NeuroTrack package from the IncuCyte software.

1.4. Preparation of total DNA for HPLC.

Cells were washed in PBS, lysed in lysis buffer (10 mM Tris HCl [pH 7.4], 50 mM NaCl, 0.5% SDS, 100 mM EDTA, 300 µg/ml proteinase K) and incubated at 50°C for 2 hours. Total nucleic acid was recovered from the completely lysed sample by ethanol precipitation in 2 volumes of 100% ethanol at room temperature (for 30 minutes), and pelleted by centrifugation. The pellet was washed once in 2 volumes of 70% ethanol, and the nucleic acid pellet was resuspended in hydrolysis buffer containing 1x DNase I buffer (*NEB*), 1mM zinc sulphate, DNase I (*NEB*) and Nuclease P1 (*Sigma*). After 4 hours, the sample was mixed thoroughly and digested for a further 8 hours. After 12 hours at 37°C, the sample was heated to 92°C for 3 minutes and cooled on ice. Two volumes of 30 mM sodium acetate, 1 mM zinc sulphate [pH 5.2], Nuclease P1 were added, and nucleic acids were digested to deoxyribonucleotide and ribonucleotide 5' monophosphates by incubating for 24 hours at 37°C.

1.5. HPLC quantification of nucleotide content.

HPLC was performed on the 5 µm Apex ODS C18 column, with isocratic 50 mM ammonium phospate (monobasic) mobile phase. UV absorbance was recorded at 276 nm (dCMP, elution time 9.4 minutes), 282 nm (5mdCMP, elution time 17 minutes), 268 nm (dTMP, elution time 21.9 minutes), 260 nm AMP and dAMP (elution times 27 minutes and 62.47 minutes) and 254 nm (GMP and dGMP, elution times 11.1 minutes and 29.7 minutes). Extinction coefficients used in nucleotide quantifications were dCMP, 8.86 x 10^3; 5mdCMP 9.0 x 10^3; dTMP, dGMP/GMP 12.16 x 10^3; dAMP/AMP 15.04 x 10^3. Relative amounts of all nucleotides were calculated from the area under each peak (Chromeleon software) using the respective extinction coefficients.

1.6. Methylation-dependent repression assay.

Repression assay was performed using Dual Luciferase assay kit (Promega) according to manufacturer's protocol. First, we inserted Firefly luciferase containing CpGs into CpG-free plasmid obtained from InvivoGen. 100 µg of CpG-free luciferase plasmid was methylated with 200 U M.Sss I (NEB) in the presence of SAM for 2 h 40 min at 37°C. Simultaneously, the same amount of the plasmid was incubated in the buffer with SAM but without M.Sss I to be used as unmethylated control. Reaction was deactivated at 65°C for 20 min. Plasmids were purified from proteins by PCI (Sigma) and DNA was precipitated from the water phase using isopropanol. To confirm the methylation status of the plasmids, restriction enzymes Hpa II and Msp I (NEB) were used. CpG-free luciferase plasmid, plasmids containing human MeCP2 either WT or R111G or R306C and plasmid containing *Renilla* luciferase were transfected into *MBD2-/- MeCP2 -/y* MEFs (total amount transfected: 500 ng). Specifically, 5 ng of unmethylated or methylated CpG-free luciferase plasmid was mixed with 500 ng of CpG- and luciferase-free plasmid, and further mixed with 100 ng of plasmid expressing MeCP2 and 12.5 ng of plasmid expressing *Renilla* luciferase. This mixture of DNA was combined with 3.5 µl of Lipofectamine 2000 (Thermo Scientific) in the OPTIMEM medium and added onto MEFs seeded on the day before transfection at the density of 50,000 cells per well of a 24-well plate. The transfection mixture was incubated with cells at 37°C for 5-6 hours and after that the medium was changed. Dual luciferase assay was performed 48 h after transfection as follows. Transfected cells were lysed in 1x Passive Lysis buffer at RT for 15 min with gentle rocking. 100 µl of Luciferase substrate were transferred into an illuminometer tube. 20 µl of lysed cells were added and luminescence of the firefly luciferase was recorded. Next, immediately after the first measurement of the firefly luciferase, Stop and Glo reagent was added and *Renilla* luciferase activity was measured. All measurements were done in at least three replicates and ratios of the Firefly and *Renilla* luciferase activities were calculated for unmethylated and methylated plasmids.

2. Library preparation for Next Generation Sequencing.

2.1. TAB-seq.

The TAB-treated genomic DNA was sonicated for 30 cycles of 30 sec ON and 30 sec OFF on low power using Bioruptor (Diagenode). DNA was end-repaired and the ends were 3'-adenylated in order to facilitate adapter ligation. Size selection was performed using Agencourt AMPure XP (Beckman Coulter) beads. After adapter ligation and size selection, DNA was treated using the EpiTect Bisulfite kit (Qiagen) and PCR amplified using custom primers. All

libraries were sequenced as 100 bp long pair-end reads on HiSeq 2500 Illumina platforms. Raw data were deposited in GEO database (accession number GSE125660).

2.2. RNA-seq.

Total RNA was isolated from all generated cell lines (Table S1) at day 9 of differentiation using either the RNeasy Mini kit or the AllPrep DNA/RNA Mini kit (Qiagen). Genomic DNA contamination was removed with the DNA-free kit (Ambion) and remaining DNA-free RNA was tested for purity using PCR for the *GAPDH* genomic locus. Total RNA was tested on the 2100 Bioanalyzer (Agilent Technologies) to ensure a RIN quality higher than 9, and quantified using Nanodrop. Equal amounts of total RNA were taken forward for library preparation and ERCC RNA Spike-in control mixes (Ambion) were added according to the manufacturer's guide. Ribosomal RNA was depleted using the Ribo-Zero Gold rRNA Removal module (Epicentre, Illumina). Isolated mRNA was tested for purity on the 2100 Bioanalyzer. mRNA was quantified using Qubit and equal amounts of each sample were used for cDNA synthesis and 3' terminal tagging using ScriptSeq v2 RNA-seq library preparation kit (Epicentre, Illumina). Libraries were PCR-amplified to add adaptors and barcodes. The libraries were sequenced as 100 bp pair-end reads using HiSeq 2000 or HiSeq 2500 Illumina platforms. Raw data were deposited in GEO database (accession number GSE125660).

2.3. ATAC-seq.

Neurons were scraped from the plate and nuclei were isolated using a hypotonic buffer (10 mM Tris-HCl pH7.4, 10 mM NaCl, 3 mM $MgCl_2$, 0.1% [v/v] Igepal CA-630), and counted. 50,000 nuclei were resuspended in 50 µl of a transposition reaction mix containing 2.5 µl Nextera Tn5 Transposase and 2x TD Nextera reaction buffer. The mix was incubated for 30 min at 37 ºC. DNA was purified by either the MinElute PCR kit (Qiagen) or the Agencourt AMPure XP (Beckman Coulter) beads and PCR amplified with the NEBNext High Fidelity reaction mix (NEB) to generate DNA libraries. The libraries were sequenced as 75 bp long pair-end reads on a HiSeq 2500 Illumina platform. Raw data were deposited in GEO database (accession number GSE125660).

2.4. MeCP2 ChIP-seq.

LUHMES-derived neurons at day 9 of differentiation with four levels of MeCP2: KO, WT, OE 4x and OE 11x (Table S1) were crosslinked with 1% of Formaldehyde (Sigma) for 10 min at room temperature (RT) and quenched with 2.5 M Glycine (Sigma) for 2 min at RT. Cells were washed with PBS, scraped from the plate and centrifuged. Crosslinked nuclei were isolated in a hypotonic buffer (10 mM Tris-HCl pH7.4, 10 mM NaCl, 3 mM $MgCl_2$, 0.1% [v/v] Igepal CA-630) and counted using a haemocytometer. Chromatin from ~$4x10^6$ nuclei was sonicated for

20 cycles (30 sec ON and 30 sec OFF) using Bioruptor (Diagenode) on high power. Crosslinked and sonicated chromatin was mixed with 60 ng of sonicated *Drosophila* chromatin (Active Motif) as a spike-in, and the mix was incubated overnight at 4 ºC with antibodies against MeCP2 (D4F3, Cell Signalling) plus spike-in antibody (Active Motif). After overnight incubation, magnetic Protein G coated beads (Thermo Scientific) were added and incubated for 4 hours at 4 ºC. Beads were washed, and chromatin was reverse-crosslinked overnight at 65 ºC. DNA was purified using the Agencourt AMPure XP (Beckman Coulter) beads. For ChIP-seq library preparation, IPs for each condition were pooled together to achieve 5 ng total DNA as a starting material. For example, 3-4 IPs were pooled together for the KO sample and 2 IPs were pooled for the WT sample. Libraries were prepared using the NEBNext Ultra II DNA library Prep kit (NEB) for both IPs and corresponding inputs. The libraries were sequenced as 75 bp long pair-end reads on a HiSeq 2500 Illumina platform. Raw data were deposited in GEO database (accession number GSE125660).

3. Bioinformatics and data preparation.

3.1. Bisulfite sequencing (TAB-seq).

Trimmomatic version 0.32 (1) was used to perform quality control on paired-end reads to remove adapter sequence and poor-quality bases at the ends of reads for both BS-sq and ChIP-seq. For BS-seq, we used Bismark version 0.10 (2) to further align and process the reads. Mapping was performed in bowtie2 mode to the human hg19 reference genome. Following alignment, reads were deduplicated and methylation values were extracted as bismark coverage and cytosine context files. We calculated the methylation percentage (mC%) at each cytosine position as:

mC% = (number of methylated reads (mC) / total number of reads (mC+C))*100

and generated *.bed files for further processing. We used two parameters to characterize methylation of genomic regions:

mC mean = sum of mC% for each cytosine / number of cytosines in the genomic region

mC density = sum of mC% for each cytosine / length of the genomic region.

3.2. ChIP-seq and ATAC-seq.

We used bwa mem version 0.7.5 (3) to map reads to the human hg19 reference genome. We filtered the alignments to remove reads that map to multiple locations in the genome and to blacklisted regions defined by the ENCODE project. We further removed duplicate reads with Picard version 1.107 MarkDuplicates (http://broadinstitute.github.io/picard/). To account for varying read depths we used deepTools version 2.5.1 (4) to create bigWig files normalised by RPKM (reads per kilobase per million reads). To quantify MeCP2 occupancy on the genomic features of interest (mCG, mCA, GT, etc.), we reject fragments longer than 1 kb regarding them as alignment artefacts.

3.3. RNA-seq.

All paired-end sequencing reads were trimmed, and quality controlled using Trimmomatic version 0.33 (1). The filtered reads were then mapped using STAR version 2.4.2 (5) using hg19 human genome assembly and Ensembl 74 release for annotation. Additionally, TPM values for genomic features were quantified by quasi-mapping approach using Sailfish version 0.10.0 (6). Protein coding transcripts for Sailfish index generation were taken from Gencode release 19. In order to assign reads to genomic features, featureCounts version 1.5.0 was used (7). Differential expression analysis was performed using DE-Seq (8). Mutant vs wildtype comparisons were performed within the same batch using the design formula ~differentiation+condition in order to account variance from the differentiation.

4. Transcriptome analysis.

4.1. Filtering RNA-seq data to test robustness of effect.

We attempted to eliminate genes whose Log2FC fluctuated too much among different levels of MeCP2 by selecting only such genes for which Log2FC changed quasi-monotonously across five MeCP2 levels: KO, WT, OE 3x, OE 4x, and OE 11x. Specifically, we selected genes for which the Spearman rank correlation between Log2FC and MeCP2 level was larger than 0.8. This resulted in 2252 genes (out of $\sim 17{,}000$ used in Fig. 1C). Fig. S10A shows that the number of such genes decreases quickly when an additional constraint on Log2FC>cut-off value is imposed, and only about 500 genes show a two-fold change between OE 11x and OE control.

Fig. S10B shows that plots of Log2FC versus mCG density are very similar to those for unfiltered genes (Fig. 1C). However, statistical uncertainty (characterized by error bars) is larger due to a smaller number of genes and less good averaging out of MeCP2-independent

effects. Using higher cut-off for the Spearman correlation leads to less selected genes and even higher uncertainty.

Besides selecting genes with respect to correlations between Log2FC and MeCP2 level, we tried other approaches to remove the noise in Fig. 1C:

- Select only statistically significant genes (based on p-values returned by DE-Seq, the software used to generate Log2FC from RNA-seq) (Fig. S4A). Even setting aside problems with this approach (Log2FC known only up to an additive constant), this approach did not significantly change the results, apart from reducing the number of analysed genes.

- Select only genes with high/low TPMs. We observed no change except larger error bars due to smaller number of genes in each bin.

- Select only long genes (>100kb). This increased the slope of Log2FC vs mCG density approximately two-fold (see Fig. S4B for plots for KO/WT and OE 11x/OE ctr). We note that this is what we would expect from our congestion model with slow-sites, which predicts that longer genes should be affected more strongly.

4.2. Maximum slope calculation.

To obtain the slope (Fig. 1D) we used an automated algorithm for each cell line. We fitted straight lines $\mathrm{Log2FC} = a\rho_{\mathrm{mCG}} + b$ to Log2FC(expression) to different ranges of $\rho_{\mathrm{mCG}} \in [\rho_1, \rho_1 + \Delta\rho]$, for different pairs $\rho_1 \in [0.5,1]$ and $\Delta\rho \in [1,4]$ (units: 1/100bp). The maximum slope was taken to be equal to a of the fit with the largest absolute slope-to-error ratio (largest $|a|/\sigma_a$ where σ_a is the standard error of a). While the algorithm sometimes fails to find the true maximum slope, we prefer to use it instead of manual fitting to minimise a possible cognitive bias.

The maximum linear slope is a good measure of the profile's steepness because it is sensitive to the level of MeCP2, and does not depend on absolute values of Log2FC. We also tried non-linear fits (exponential function or a polynomial) but since such fits require more than one parameter, selecting a suitable combination of parameters that would give something akin to a "slope" was ambiguous and did not improve the results compared to the linear fit.

4.3. Possible explanations of Log2FC increase in the OE with mCG density for $\rho_{mCG} < 0.8$.

Figure 1C shows a non-monotonous behaviour of Log2FC(OE 11x/OE ctr) as a function of mCG density: Log2FC initially increases with mCG, peaks at about 0.8/100bp, and then decreases with mCG density. In the main text, we focus on the range 0.8-4/100bp which contains most of the expressed genes. Below we discuss possible explanations of the low-mCG behaviour.

i) **Activation at low mCG densities.** It is possible that the mechanism of MeCP2-dependent transcription regulation is different for low mCG densities. We considered the following hypothesis: low gene-body methylation (0… 0.8/100bp) increases transcription in the OE compared to KO because additional MeCP2 helps the chromatin to stay in the open state. This accounts for the increase in Log2FC for low mCG density. However, if too much MeCP2 is bound to the DNA (higher mCG density), MeCP2 blocks transcription due to Pol II queuing as discussed in the manuscript. This accounts for the falling slope of the Log2FC curve for mCG density larger than approx. 1 per 100bp. At an mCG density close to 1/100bp the two effects balance each other and we get a maximum of Log2FC in the OE 11x. Regardless of the mechanistic details and assuming that transcription is affected by the density of MeCP2 (and not just mCG density), this hypothesis would predict that Log2FCs for different MeCP2 levels should collapse onto a single curve when plotted as a function of MeCP2 occupancy on genes. This implies that, when plotted as a function of mCG density (Fig. 1C), the position of the peak in the OE 11x (at approx. 1/100bp) should shift to the right (approx. position: 3/100bp) in the OE 3x. We do not observe this; in fact, the position of the peak in all OEs remains close to 0.5-1/100bp.

ii) **Artefact.** It is possible that this is an artefact and not a genuine effect. There are only 726 genes (4.7% of all analysed genes) in this range; 87% genes have mCG density between 0.8 and 4 per 100bp. Log2FCs may be strongly affected by gene-gene interactions which have not been averaged out due to the small number of genes, and therefore the results may not truly reflect the “average”, MeCP2-dependent behaviour.

5. ChIP-seq accumulation algorithm.

For each chromosome we create an array n_i of the number of instances a particular locus (a single base pair at position i in the chromosome) is covered by a ChIP-seq read. We do this by going through all ChIP-seq reads from *.bed files, each time incrementing all n_i's for which i is between the start and end position of a given read. When all reads in a given chromosome have been processed, for each feature x ($x =$ mCG, non-mCG, …) at position j we select a region of interest of length L around it ($j \pm L/2$ bp) and accumulate the counts in a separate array (different for each feature x): $c_i^x \to c_i^x + n_{i+j}$, for all i such that $-\frac{L}{2} \leq i < \frac{L}{2}$. The obtained

accumulated counts c_i^x for ChIP-seq are divided by the accumulated counts for the corresponding input DNA-seq to reduce the sequencing bias present in the data.

To demonstrate the above procedure, let us take the following sequence as the reference genome with features of interest (in this case CG) marked red:

```
aaacagctagttaattttgaatcgcaggtaaacaatcgaataattttcta
```

We assume that ChIP-seq has generated the following reads:

```
              ttttgaatcgca
                   aatcgcaggta
             attttgaatcg
                      cgcaggtaa
                                caatcgaa
                                   tcgaataattt
```

The number of reads n_i covering a specific site in the genome is

```
00000000000001222223334433222210111222221111110000
```

Raw counts over a fixed-length region (here +/-5bp, in the actual algorithm this would be +/-5000bp) centred at the feature of interest are

```
223334433222
011122222111
```

Total accumulated counts c_i^{CG} versus position i relative to the feature of interest are therefore

```
234456655333
```

In the actual analysis, the above sequence would be a 10 kb-long array of integers.

6. Details of the ChiP-seq computer model.

6.1. The algorithm.

The input to the simulation are the *.bed files with ChIP or input (DNA) reads, and the two parameters p, p_{bg}. The simulation uses the data files to preserve the distribution of the lengths of reads but not their positions. The positions are determined by the following algorithm:

1) For each input file, GC content- and length bias is first estimated by constructing a table of counts $r[\%GC][l]$ for the actual reads and $g[\%GC][l]$ for genomic sequences of the same length l, %GC, and randomly selected locations.

2) For each read from the experimental *.bed file we calculate its length $l = e - s$ and then select a random start point s (within the same chromosome as the original read) and the end point $e = s + l$.
3) GC content and the number of mCs for the simulated read is calculated. We take a particular C to be methylated with probability proportional to the fraction of methylated reads containing this site in our methylation data (TAB-seq).
4) If there is at least one mC within the read, an auxiliary variable P is set to 1 with probability p times the relative binding affinity of the motif to which this C belongs. Otherwise P is set to p_{bg}. This accounts for MeCP2 present/absent in this particular DNA fragment.
5) P is multiplied by $r[\%GC][l]/g[\%GC][l]$ to account for the GC content and read length bias.
6) The simulated read is accepted with probability P and saved to a file, or rejected (with probability $1 - P$). If the read is rejected, another random position s is selected and the algorithm continues from (3).
7) Go to step 2 unless all reads from the *.bed file have been processed.

Simulated reads are then processed in the same way as the experimental ChIP data (SI Section 5).

6.2. Height of the ChIP-seq enrichment peak.

Since mCG is much more frequent than any other MeCP2-binding motif in our cell lines, in what follows we focus on enrichment profiles on mCG. Fig. S11A shows simulated enrichment profiles on mCG obtained for $p_{bg} = 0$ and different p. Counterintuitively, the height of the central peak does not decrease with decreasing p, but it slowly increases. The height of the peak tends to a constant as $p \to 0$ (Fig. S11B). This can be explained as follows. For large p, fragments centred at adjacent mCGs overlap, increasing the counts c_i^{mCG} in the flanking regions ($|i| \gg 1$). Normalization lowers the apparent height of the peak since it divides the profile by the counts in the flanking regions. As p decreases, the number of overlapping fragments from neighbouring mCGs decreases; this reduces raw counts in the flanks and increases the relative height of the central peak after normalization.

6.3. Fitting ChIP-seq to data.

For each ChIP-seq data set we fitted the simulated profile f_i^{sim} (parametrized by p, p_{bg}) to the experimental enrichment profile f_i^{expr} by minimizing the sum of squared differences,

$$\chi^2 = \sum_{i=-100}^{100} \left(f_i^{expr} - f_i^{sim}\right)^2,$$

with respect to p and p_{bg}. The region +/-100bp was chosen because of the sensitivity of the profile shape to p, p_{bg} in this region. Minimization was performed as follows: we simulated profiles for a range of p and two values of p_{bg}: 0 and 0.9; profiles for p_{bg} between these two values were obtained by linear interpolation (permitted due to the assumed additive model of signal and background reads). The minimum χ^2 obtained in this way for 11x OE is plotted in Fig. S6D. Any $p \leq 0.1$ gives similar (low) values of χ^2, indicating that $p \approx 0.1$ is the upper bound on mCG occupancy in 11x OE.

A peak is noticeable in KO (Fig. 2B) where MeCP2 is absent. We think the peak may be caused by non-specific binding between proteins other than MeCP2 and antibodies used in the immunoprecipitation. Even a minute amount of binding ($p \ll 1$) by an MeCP2-mimicking protein can create a peak. CG and length bias (different in different replicates and impossible to completely remove) probably also contributes to the peak. Non-uniform profiles (peaks or troughs) are present in virtually any ChIP-seq even in the absence of the protein of interest, but they are usually not shown; instead, a common practice is to divide the profile of interest by the “control” profile (KO in our case). We decided to present the KO ChIP-seq data to show our confidence in the ChIP/MeCP2 binding model which is able to reproduce the peak.

To predict profiles on unmethylated CG (Fig. S6E), we used the values of (p, p_{bg}) obtained from the mCG profiles by minimizing χ^2 with respect to p_{bg} and assuming $p = 0.1, 0.05, 0.02, 0.002$ for OE 11x, OE 4x, WT, and KO, respectively. These values are based on the best-fit $p = 0.1$ obtained for OE 11x, the other p’s being a fraction of this value approximately proportional to the relative abundance of MeCP2 in a given cell line compared to OE 11x. The predicted profiles match the data very well but are not sensitive to the exact value of p. This indicates that ChIP-seq alone cannot be used to estimate the occupancy p besides providing the upper bound on p.

The dip visible in the profiles in Fig. S6E is caused by MeCP2 binding to methylated CGs surrounding the unmethylated site. This increases the number of immunoprecipitated

fragments in the flanks compared to the centre (at or near the unmethylated CG). Since all profiles are normalized by enrichment in the flanks, the centre looks as if it was rarefied.

7. ATAC-seq model.

7.1. The algorithm.

We simulate binding to a short DNA sequence (first L=50Mbp of chromosome 1). We assume that MeCP2 occupies 11bp (20, 21) and that the protein is centred on an mC, thus the obscured genomic sequence is xxxxxmCxxxxx where x can be any nucleotide.

The following algorithm is repeated T times (larger T corresponds to longer digestion times in the experiment):

1) A random location i is chosen as the position of the new cut. We assume the following convention: i refers to the position of the nucleotide immediately to the left from the centre of the cut. Hence, $i+1$ denotes the position of the nucleotide to the right from the cut. Positions $i, i+1$ are the positions of insertion sites.
2) The position is accepted with probability p_i which depends on the nucleotide sequence $i-w, \dots, i+w$ where w=10 bp. This is based on the known Tn5 sequence preference across $21 = 2w+1$ bp that it contacts (9, 10). The probability p_i is calculated using the position weight matrix (PWM) obtained from ATAC-seq reads for KO1.
3) If the position is rejected, go to step 1 and try again.
4) If there is enough space for Tn5, a cut is made between i and $i+1$. We assume that Tn5 occupies 21bp ($i-w, \dots, i+w$) and for a cut to be made there must not be any protein (MeCP2) overlapping with this region. There must not be any previous cuts made in this region too, otherwise the proposed cut is rejected.
5) If the proposed cut is rejected, go to step 1 and try again.
6) A weight $W = \exp(b \times GC)$ is assigned to each fragment where GC is the GC content (0…1) of the fragment and b is a constant. This simulates an additional GC bias that may be created during library preparation and DNA-sequencing procedures.

The shortest possible fragment generated by the algorithm is 21bp which corresponds to two Tn5 cutting just next to each other. Since MeCP2 occupies 11bp, the shortest MeCP2-containing fragment is 11+21=32bp. We retain only fragments longer than 35bp because our experimental data does not contain fragments shorter than this.

The simulation creates artificial fragments that we process in the same way as the experimental data. The input to the program is the DNA sequence (fixed), the density p of

MeCP2 on mCxx, the average density of insertion (cut) sites $t = T/L$ (cannot be larger than 1/21bp), and the GC bias b.

7.2. Algorithm benchmarking

To test the role of the parameters on the shape and depth of the simulated footprint of MeCP2, we run the model for different p, t, b. We also performed simulations with/without Tn5 bias.

Fig. S12A shows the counts profiles for $p = 0$ (no MeCP2) with and without Tn5 bias, compared to the experimental profile. It is evident that the insertion bias is required to reproduce the experimental insertion profile. However, the bias cancels out when calculating the relative profile (footprint) f_i. This is demonstrated in Fig. S12B which shows the footprint obtained by dividing the counts profile for $p = 0.05$ by the profile for $p = 0$, for different t and $b = 6$ (Tn5 bias) or $b = 0$ (no bias). Increasing digestion time t increases the height of the side peaks surrounding a depression caused by MeCP2. However, the depth of the depression does not change noticeably.

Fig. S12C, left shows that the GC bias b can does not affect the depth of the footprint if the bias is the same in the simulated test and reference samples. However, the footprint is affected if the GC bias is different in the two samples (Fig. S12C, right). Since such a mismatch in the GC bias would have a notable signature (rising/falling flanks >50bp away from mCG) which we do not see in our data, we conclude that the bias is very similar in all experimental samples.

Fig. S12D shows that dividing the counts for $p > 0$ by the counts for $p = 0$ but with a different digestion time t changes the depth of the footprint slightly. Experimental variation causes t to be slightly different for different samples, and hence our results can have a small systematic error.

Our simulations cannot explain two subtle features of the experimental data: the presence of oscillations superimposed on the main profile, and a small central peak visible in WT/KO, and OE 4x/KO. We speculate that the first is caused by steric interactions between MeCP2 and Tn5, and the latter by interactions of proteins other than MeCP2 with mC or a small difference in the GC bias among the samples.

7.3. Fitting simulated ATAC-seq profiles to data.

We used the depth of the footprint to extract MeCP2 occupancy p. We simulated ATAC-seq for many pairs (p, t), for $p = 0 \dots 0.1$, $t = 0 \dots 0.08$, and a fixed $b = 6.0$ (the exact value is not important since GC bias cancels out when calculating f_i). We then selected those (p, t) which minimized the distance between the simulated and experimental footprints f_i^{sim} and f_i^{exp},

$$D = \sum_{i=\{-9,-10,14,15\}} \left(f_i^{exp} - f_i^{sim}\right)^2 \times \sum_{i=-100}^{100} \left(f_i^{exp} - f_i^{sim}\right)^2.$$

This formula assigns a larger weight to the edges of the central dip which is the region least sensitive to variations in the ATAC-seq protocol, and reduces the systematic error caused by the (small) central peak of unknown origin (SI Section 7.2). The best fit to the 11x OE footprint yields $p = 0.063, t = 0.04$. Fig. 2E shows p for all MeCP2 levels M. The relationship is linear, with the best-fit $p = 0.0058 \times M_{cell\ line}/M_{WT}$.

8. Generic model of gene expression.

Below we list all the parameters and observables of the generic ("paradigm") model of gene expression (22) from Fig. S7A which is the starting point for all other models. For each gene i we define

- $k_{i,ON}, k_{i,OFF}$ – the switching rates OFF→ON and ON→OFF, respectively.
- J_i – transcription rate (in transcripts/second).
- α_i – transcription initiation rate (1/second)
- v_i - transcription elongation rate (bp/second).
- d_i – mRNA degradation rate (1/second).
- f_i – the fraction of cells in the population that at any given time have gene i in the ON (actively transcribed) state.

We assume that mRNA is degraded according to 1st order kinetics (11). The steady-state concentration c_i of mRNA from gene i is obtained by equating production (transcription in the ON state) and degradation rates:

$$J_i f_i = c_i d_i, \qquad \text{(Equation S1)}$$

from which it follows that

$$c_i = J_i f_i \,/d_i. \quad \text{(Equation S2)}$$

The number of transcripts per million (TPM) which we obtain from RNA-seq is thus

$$\mathrm{TPM}_i = \; N J_i f_i \,/d_i, \qquad \text{(Equation S3)}$$

where N is an unknown proportionality constant that depends on the sample and is different for different cell lines, replicates and conditions but is the same for all genes in a given sample.

9. Condensation model.

This model considers a hypothesis (ultimately proven to be false, see the main text) that MeCP2 causes chromatin condensation which reduces the fraction of cells with genes in the active (ON) state (Fig. 3A). We assume that the fraction f_i of cells with gene i in the active state depends on promoter openness a_i (measured by ATAC-seq) which in turn depends on the level M of MeCP2 and gene methylation ρ_i:

$$f_i = f_i(M, \rho_i) \propto a_i = a_i(M, \rho_i). \qquad \text{(Equation S4)}$$

The model also assumes that transcription rate J_i and mRNA degradation rate d_i are not affected by MeCP2. The Log2FC of differential expression of gene i for cell lines X and Y is then

$$\mathrm{Log2FC}_{\mathrm{X/Y},i} = \log_2 \frac{\mathrm{TPM}_i(\mathrm{X})}{\mathrm{TPM}_i(\mathrm{Y})} = \log_2 \frac{N_\mathrm{X}}{N_\mathrm{Y}} + \log_2 \frac{a_i(M_\mathrm{X}, \rho_i)}{a_i(M_\mathrm{Y}, \rho_i)}.$$

The average Log2FC of genes with the same methylation density ρ is therefore

$$\mathrm{Log2FC}_{\mathrm{X/Y}}(\rho) = \log_2 \frac{N_\mathrm{X}}{N_\mathrm{Y}} + \langle \log_2 \frac{a_i(M_\mathrm{X}, \rho)}{a_i(M_\mathrm{Y}, \rho)} \rangle,$$

where $\langle \ldots \rangle$ denotes averaging over genes with the same ρ. According to the above equation, $\mathrm{Log2FC}_{\mathrm{X/Y}}$ should yield the same curve (modulo a vertical shift due to different normalization factors N_X and N_Y) as the logarithm of the ratio of accessibilities of X versus Y when plotted as a function of methylation density. Fig. 3C shows that this is not the case. We can therefore reject the hypothesis that MeCP2 modulates gene expression primarily by altering the fraction of active genes.

10. Detachment model.

In this hypothetical scenario we assume that RNA Pol II aborts transcription with some small probability λ when it collides with MeCP2 or encounters a chemical mark left by the interaction

between MeCP2 and chromatin. The probability that RNA Pol II reaches the end of the gene (transcription end site, TES) is thus

$$P = (1-\lambda)^n \cong e^{-\lambda n},$$

where n is the number of “abort sites” on the gene, proportional to the number of MeCP2 molecules on the gene. ATAC-seq shows that the density of MeCP2 on a gene is proportional to its methylation density ρ and the total concentration M of MeCP2 in the nucleus, hence we can write that $n = AM\rho L = AMN_{\mathrm{mCG}}$, where A is an unknown proportionality factor, L is the length of the gene, and N_{mCG} is the total number of mCGs. Log2FC of the differential expression X versus Y can then be written as

$$\mathrm{Log2FC}_{\mathrm{X/Y}} = \log_2 \frac{\mathrm{TPM}_i(\mathrm{X})}{\mathrm{TPM}_i(\mathrm{Y})} = \log_2 \frac{N_{\mathrm{X}}}{N_{\mathrm{Y}}} + \log_2 \frac{P(M_{\mathrm{X}}, N_{\mathrm{mCG}})}{P(M_{\mathrm{Y}}, N_{\mathrm{mCG}})}$$

$$= \mathrm{const} + \log_2 \frac{\exp(-\lambda A M_{\mathrm{X}} N_{\mathrm{mCG}})}{\exp(-\lambda A M_{\mathrm{Y}} N_{\mathrm{mCG}})}$$

$$= \mathrm{const} - \gamma\, \Delta M_{\mathrm{X/Y}} N_{\mathrm{mCG}},$$

where $\gamma = \lambda A M_{\mathrm{Y}} / (\ln 2)$ is an unknown parameter identical for all cell lines, and $\Delta M_{\mathrm{X/Y}} = \frac{M_{\mathrm{X}}}{M_{\mathrm{Y}}} - 1$ is the relative difference between the level of MeCP2 in cell lines X and Y. For example, $\Delta M_{\mathrm{KO/WT}} = -1$ and $\Delta M_{\mathrm{11x\ OE/WT}} = 10$. $\mathrm{Log2FC}_{\mathrm{X/Y}}$ should therefore follow a straight line when plotted versus N_{mCG}, and the slope of this line should be positive for KO/WT, and negative (and 10x more steep) for 11xOE/OE ctr. Fig. 3E shows that when the model is fitted to the KO data to fix the unknown constant γ, it fails to reproduce the OE 11x data. We hence conclude that there is no evidence that MeCP2 causes premature termination of transcription.

11. Congestion models.

11.1. General considerations.

We consider a hypothesis that MeCP2 slows down the elongation step of transcription by creating queues of RNA Pol II in front of MeCP2 or chemical modifications left by it in gene bodies (Figs. 4A and S8A). We assume that the density of obstacles, or “slow sites”, is proportional to the density of molecules of MeCP2 bound to the DNA. We first show that a general class of models based on these assumptions is consistent with our experimental data. We then discuss two specific examples in Secs. 11.2 and 11.3.

We show in the main text that (i) the density m of MeCP2 on the DNA is proportional to the concentration M of MeCP2 in the cell and (ii) that m is proportional to mCG density ρ. We can thus write that the transcription rate J averaged over many genes with the same mCG density ρ is

$$J = J(M\rho, \rho).$$

The first argument ($M\rho \approx$ MeCP2 occupancy on the DNA) represent MeCP2-dependent modulation of transcription. The second argument of J accounts for non-MeCP2 but mCG-density dependent modulation. Fig. S13A show gene expression (TPMs) versus gene body methylation for KO and OE 11x. The difference between the two cells lines is very small, and non-MeCP2 dependent component of J dominates the behaviour of $J(\rho)$. We are thus permitted to rewrite J in the factorized form

$$J \approx [1 - \epsilon(M\rho)]K(\rho),$$

where K is a fast-changing function of ρ and a small correction $\epsilon(m)$ due to MeCP2 is a slowly increasing function of m, and $\epsilon(0) = 0$. This leads to the following expression for the Log2FC of cell lines X versus Y:

$$\text{Log2FC}_{\text{X/Y}}(\rho) = \log_2 \frac{\text{TPM}_i(\text{X})}{\text{TPM}_i(\text{Y})} \approx \log_2 \frac{N_\text{X}}{N_\text{Y}} + \log_2 \frac{[1 - \epsilon(M_\text{X}\,\rho)]K(\rho)}{[1 - \epsilon(M_\text{Y}\rho)]K(\rho)}$$

$$= \text{const}(\text{X}, \text{Y}) + \log_2[1 - \epsilon(\rho M_\text{X})] - \log_2[1 - \epsilon(\rho M_\text{Y})$$

$$\approx \text{const}(\text{X}, \text{Y}) - \epsilon(\rho M_\text{X}) + \epsilon(\rho M_\text{Y}). \qquad \text{(Equation S5)}$$

The latter approximation is valid since ϵ is assumed to be small. In particular, the Log2FC of any cell line X versus KO reads

$$\text{Log2FC}_{\text{X/KO}}(\rho) \approx \text{const}(\text{X}, \text{KO}) - \epsilon(\rho M_\text{X})$$

because $\epsilon(\rho M_\text{KO}) = \epsilon(0) = 0$. The Log2FC curves for different cell lines versus KO will therefore have the same shape when plotted in the variable ρM_X. It follows that the maximum slope should be proportional to M_X, which is what we observe in Fig. 1C, D. One important difference is that Log2FC plots from Fig. 1C do not assume KO as the reference but rather different control cell lines with low but non-zero level of MeCP2. We can however show that these results (in particular the linear dependence of the slope on MeCP2) remain approximately true also in this case.

We first need to make another (mild) assumption that $\epsilon(m)$ increases monotonically with m, is linear in m for small m, and saturates for large m. This can be justified retrospectively based

on the observed behaviour of Log2FC versus ρ. Both specific models from Secs. 11.2, 11.3 obey these assumptions.

From equation (S5) applied to the pair (CTR, KO) we have

$$\mathrm{Log2FC_{CTR/KO}}(\rho) \approx \mathrm{const(CTR, KO)} - \epsilon(\rho M_{\mathrm{CTR}}).$$

We can calculate $\epsilon(m)$ from this equation:

$$\epsilon(m) \approx \mathrm{const(CTR, KO)} - \mathrm{Log2FC_{CTR/KO}}(m/M_{\mathrm{CTR}}). \qquad \text{(Equation S6)}$$

This enables us to express (by combining (S5) and (S6)) the Log2FC of any cell line X versus control CTR as

$$\mathrm{Log2FC_{X/CTR}}(\rho) \approx \mathrm{const(X, CTR)} - \epsilon(\rho M_{\mathrm{X}}) + \epsilon(\rho M_{\mathrm{CTR}}) \qquad \text{(Equation S7)}$$

$$= \mathrm{const(X, CTR)} + \mathrm{Log2FC_{CTR/KO}}(\rho M_{\mathrm{X}}/M_{\mathrm{CTR}}) - \mathrm{Log2FC_{CTR/KO}}(\rho).$$

Since we assumed that $\epsilon(m)$ first increases linearly with m and then saturates, its largest slope (largest value of the derivative $\epsilon'(m)$) will be at $m = 0$. The maximum slope of Eq. (S7) will therefore occur at $\rho = 0$. We can Taylor-expand Eq. (S7) in the vicinity of this point and write

$$\mathrm{Log2FC_{X/CTR}}(\rho) \approx \mathrm{const(X, CTR)} + \rho M_{CTR}\epsilon'(0)\left(\frac{M_{\mathrm{X}}}{M_{\mathrm{CTR}}} - 1\right),$$

where $\epsilon'(0)$ is the derivative of $\epsilon(m)$ at $m = 0$. The maximum slope is therefore proportional to $M_{\mathrm{X}}/M_{\mathrm{CTR}} - 1$. This is corroborated by experimental results presented in Fig. 1C, D.

11.2. Slow sites model.

In this model, MeCP2 causes a chromatin modification that slows down the RNA polymerase II. To mathematically model this process, we use totally asymmetric simple exclusion process (TASEP) with open boundaries (12-14). A gene is represented as a one-dimensional chain of L sites. Each site is either occupied by a particle, representing RNA Pol II, or is empty. Particles enter the chain at site $i = 1$ with rate α (transcription initiation rate), move along the chain and exit at site $i = L$ with rate β. Sites can be either “fast” or “slow”. Slow sites represent mCGs affected by the interaction with MeCP2, whereas fast sites are all other sites (methylated or not). The speed of the particles is v on fast sites and v_s on slow sites. Slow sites are randomly and uniformly distributed, and their density is $\rho_s = \rho p$ where ρ is the mCG density, and p is the probability that an mCG is occupied by MeCP2 (as in the ChIP-seq and ATAC-seq models). p is taken to be 0.063 for OE 11x, and proportionally smaller for other cell lines ($p = 0.0058 M_{\mathrm{cell\,line}}/M_{\mathrm{WT}}$).

Since RNA Pol II occupies about 60bp on the DNA and moves with the speed of about 60-70 bp/s (15), it is convenient to equate a single site of the model with a 60bp-long stretch of the actual DNA, and set the RNA Pol II speed to $v = 1$ sites per second on fast sites. We also assume $\beta = v = 1$ sec^{-1} so that RNA Pol II is not blocked from exiting the chain at the end ($\alpha < \beta$ for all genes).

We simulated this model for different chain lengths $L = 166, 500, 1666, 5000$ corresponding to gene lengths between 10kb and 300kb, and a range of initiation rates $\alpha \in [0.001,1]$, densities of slow sites $\rho_s \in [1/64, 8]$ (mCG density between 0.026 and 13.3 per 100bp), and slow-site velocities $v_s = 0.01, 0.02, 0.05, 0.1, 0.2$ (all rates are in 1/sec). For each set of (α, ρ_s, v_s) we first let the model to reach steady state ("thermalization step"). We then measured the flow J of particles (equivalent to the rate of transcription) through the chain. The flow strongly depends on ρ_s and only weakly (logarithmically) on the length L (Fig. S13B and C). Therefore, in what follows we fix $L = 5000$ sites, which is equivalent to the gene length of 300 kb. The flow J obtained from these simulations is presented in Fig. S8B as a function of α, for different MeCP2 densities ρ, and for $p = 0.063$ (OE 11x). The flow is approximately linear in α until some critical α_c which depends on ρ_s, and saturates at $J = J_{max}$ when $\alpha > \alpha_c$.

To relate this model to the mRNA-seq differential expression data we must calculate Log2FC:

$$\mathrm{Log2FC}_{\mathrm{X/Y}} = \log_2 \frac{J(\alpha, \rho_{\mathrm{s,X}})}{J(\alpha, \rho_{\mathrm{s,Y}})},$$

where $\rho_{\mathrm{s,X}} = \rho p_{\mathrm{X}}$, $\rho_{\mathrm{s,Y}} = \rho p_{\mathrm{Y}}$ in which ρ_{mCG} is mCG density and $p_{\mathrm{X}}, p_{\mathrm{Y}}$ are MeCP2 occupation probabilities for cell lines X,Y. We take $p_{\mathrm{X}} = \left(\frac{M_{\mathrm{X}}}{M_{\mathrm{OE11x}}}\right) p_{\mathrm{OE11x}} = 0.05 \left(\frac{M_{\mathrm{X}}}{M_{\mathrm{OE11x}}}\right)$, and similarly for p_{Y}. In the above expression we know all quantities except the initiation rate α.

To obtain the initiation rate we bin genes according to their methylation, grouping gene with similar mCG density into bins of approximately constant width (0.1/100bp). Each of the ~80 bins is parametrized by a single value of α. Next, in each bin we find α such that Log2FC(OE 11x/OE ctr) from the above equation equals the average experimental Log2FC in the bin. This gives us $\alpha(\rho)$ as a function of methylation density ρ (Fig. S8E; average $\alpha = 0.027$ s^{-1}), which exactly reproduces the OE 11x/OE ctr data. This approach, rather than fitting initiation rates of individual genes, removes correlations due to gene-gene interactions and produces a relatively smooth curve $\alpha(\rho)$.

We can then use the fitted $\alpha(\rho)$ to predict $\mathrm{Log2FC}_{\mathrm{X/Y}}$ for other pairs of cells lines. The results are presented in Fig. S8C, D and, as described in the main text, are in good agreement with experimental Log2FCs.

A model in which J is first evaluated for individual genes (defined by their ρ, L) and then Log2FC obtained by binning genes according to their mCG density ρ_{mCG} does not significantly affect the results.

11.3. Dynamical obstacles model.

This model is very similar to the slow sites model with two exceptions: (i) polymerase always moves with the same speed v (no slow sites) as long as it is not blocked by other polymerases and obstacles, (ii) obstacles binds and unbinds dynamically from the methylated sites. These obstacles can be MeCP2, other proteins recruited by MeCP2, or structural changes induced by MeCP2. We assume that unbinding occurs with rate k_u per obstacle, whereas binding occurs with rate $k_u p$ per unoccupied mCG. An obstacle does not bind if an mCG is occupied by an obstacle or a polymerase. The parameters of the model are: α, v, L, ρ, p and, in addition, the unbinding constant k_u. Since the exact nature of obstacles in not specified, the density p of sites that bind obstacles does not have to be the same as the MeCP2 occupancy estimated from ATAC-seq data. In fact, we found that the model reproduces the data best when $k_u = 0.04$, and $p = M/M_{\mathrm{OE11x}}$, i.e., $p = 1$ for the OE 11x cell line.

The model behaves similarly to the slow-sites model. Fig. 4B shows a plot of the flow as a function of α and ρ. Fig. 4E, F shows that after fitting the initiation rates as described for the slow-site model (Fig. S8F) the model is also able to reproduce the experimental data.

The fact that the apparent fraction p of occupied mCGs must be close to 1 in OE 11x suggest that MeCP2 may slow down RNA Pol II by altering chromatin structure rather than by direct steric interference.

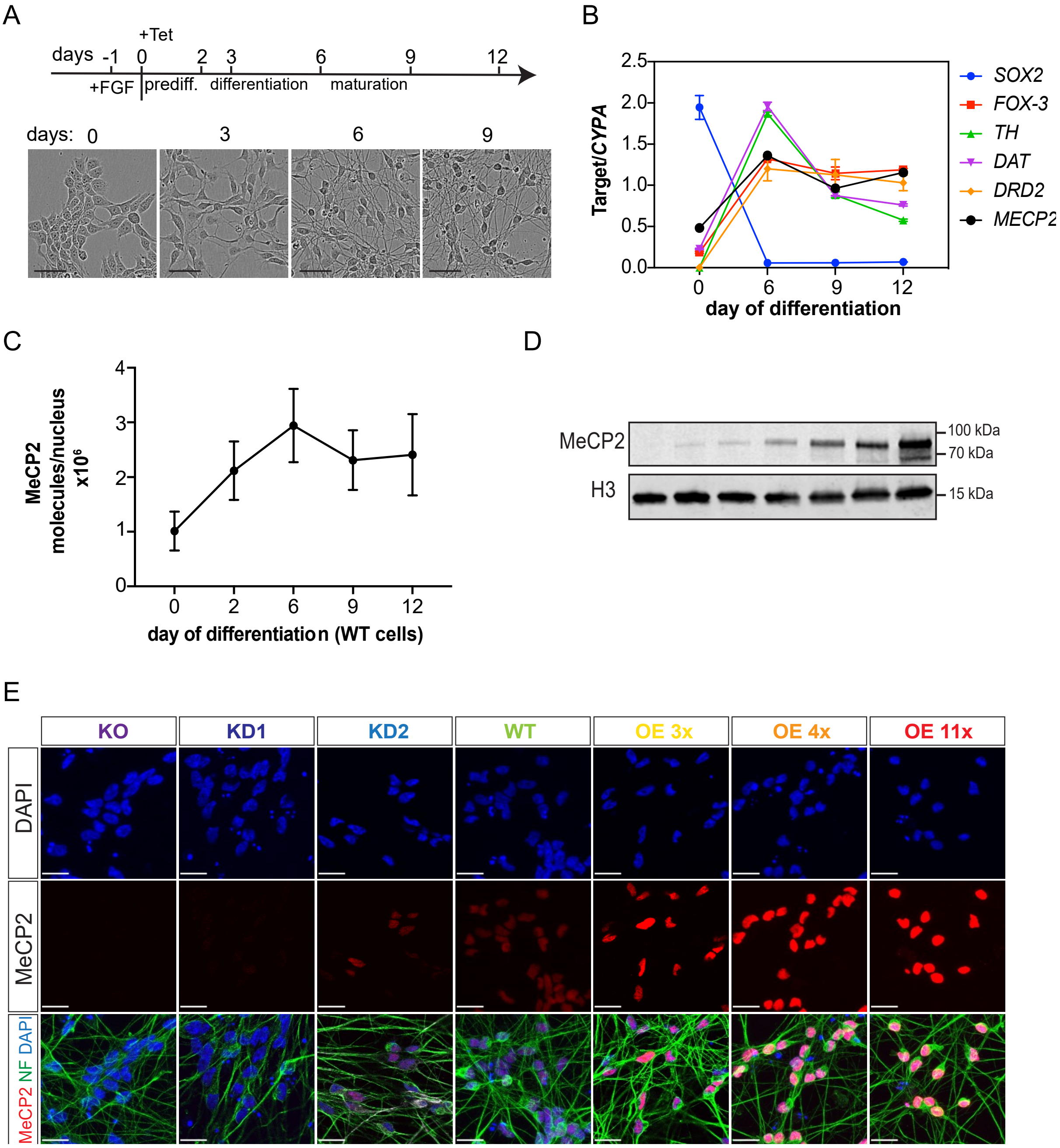

Fig. S1. Characterisation of LUHMES-derived cell lines KO and WT. (A) Experimental protocol for LUHMES differentiation. Phase contrast images show cells in different stages of differentiation. Scale bar is 50 μm. (B) Expression of neuronal differentiation markers normalized to the housekeeping gene *CYPA* during WT LUHMES differentiation. Error bars represent +/-SEM. (C) Changes in the number of MeCP2 molecules per nucleus during differentiation of WT LUHMES cells, calculated from Western blotting. Error bars represent +/-SEM. (D) Expression of MeCP2 in cells at day 9 of differentiation (Western blot). (E) Immunofluorescence imaging of MeCP2 and neurofilament (NF) expression in day-9 neurons. Scale bar is 20 μm.

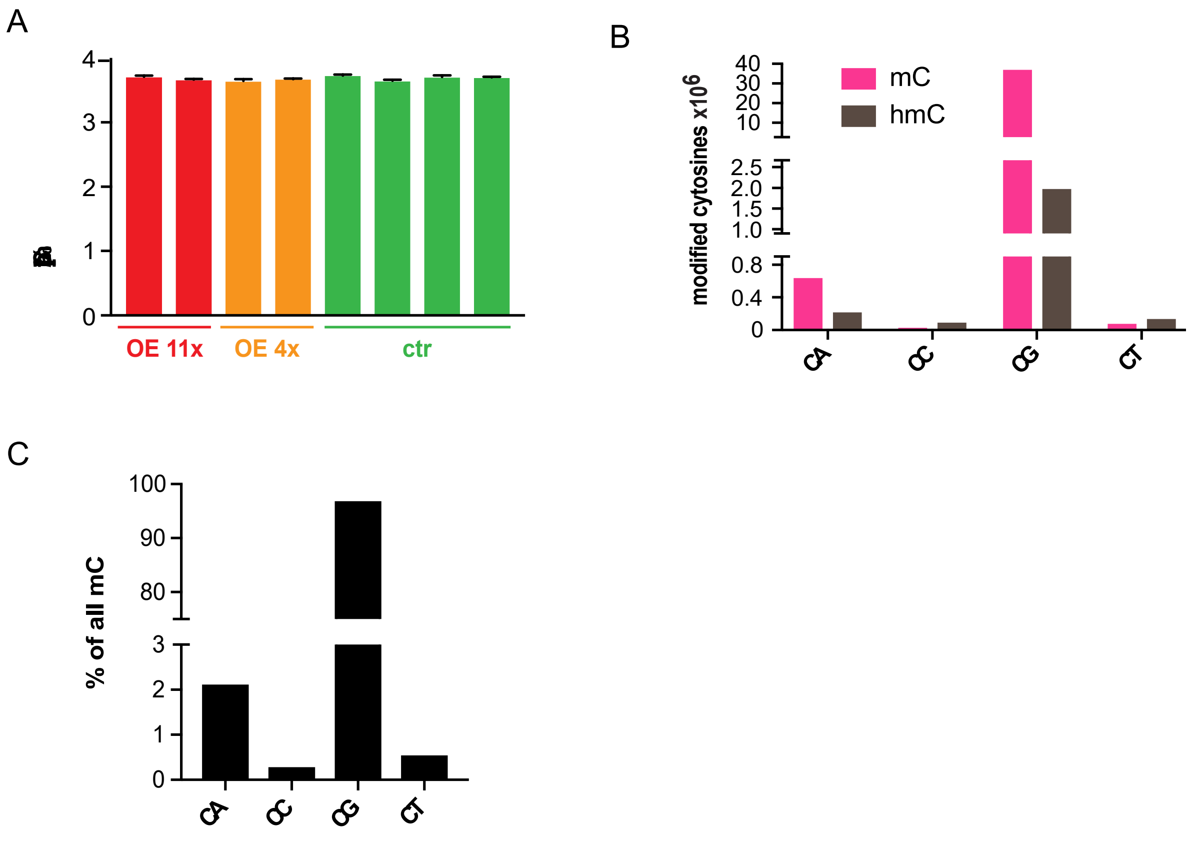

Fig. S2. DNA methylation in LUHMES WT cells. (A) Fraction of methylated Cs in the genome (quantified by HPLC) for LUHMES-derived neurons at day 9 of differentiation. OE 11x (red), OE 4x (orange) and controls (green). Error bars are +/-SEM. (B) Number of methylated (mC) and hydroxymethylated (hmC) dinucleotides (per haploid genome) obtained from TAB-seq in WT LUHMES-derived neurons (day 9). (C) Percentage fraction of methylated Cs in the context of different dinucleotides calculated from TAB-seq data.

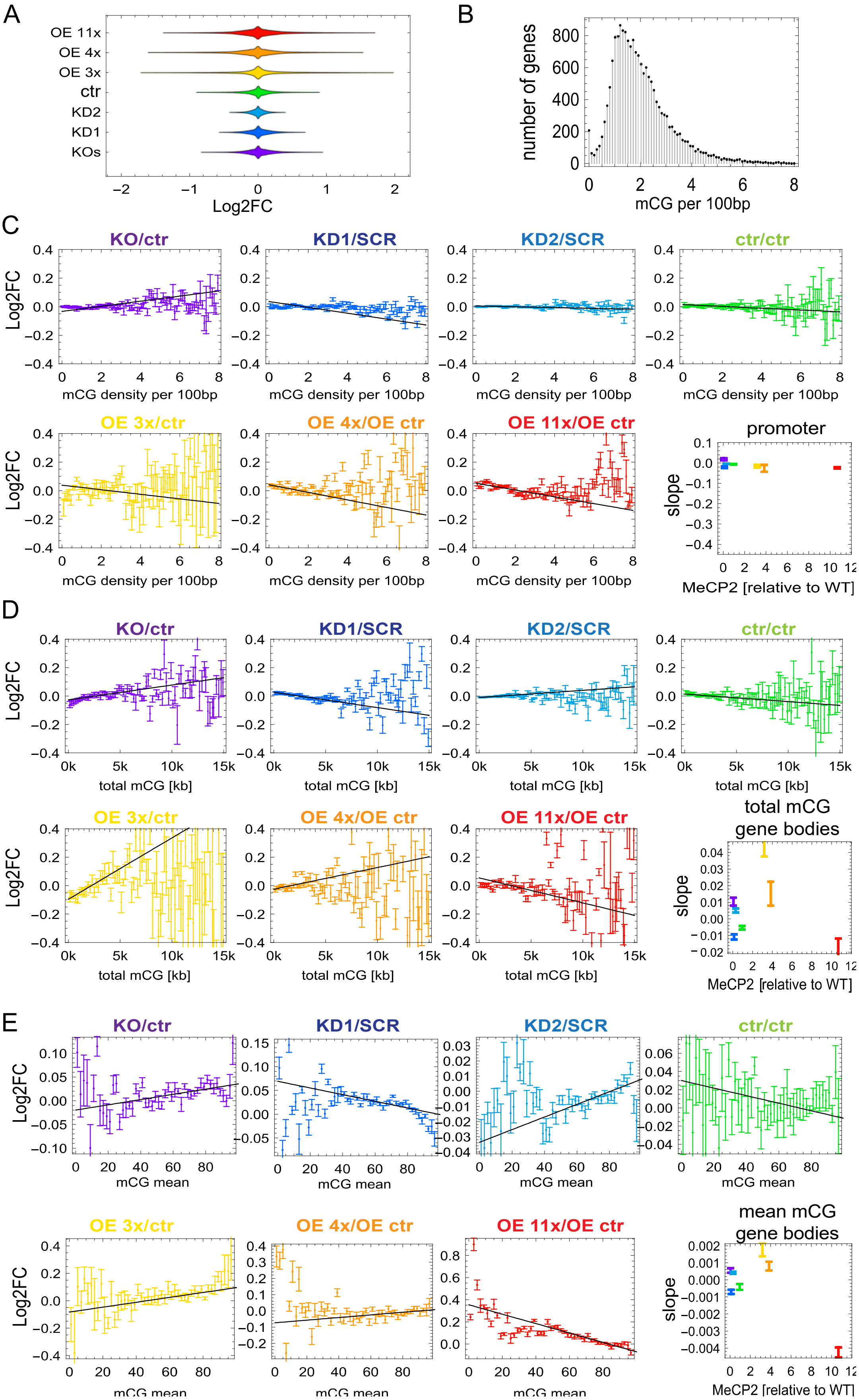

A
OE 11x
OE 4x
OE 3x
ctr
KD2
KD1
KOs
Log2FC
B
number of genes
mCG per 100bp
C
KO/ctr
KD1/SCR
KD2/SCR
ctr/ctr
OE 3x/ctr
OE 4x/OE ctr
OE 11x/OE ctr
Log2FC
mCG density per 100bp
promoter
slope
MeCP2 [relative to WT]
D
total mCG [kb]
total mCG
gene bodies
E
mCG mean
mean mCG
gene bodies

Fig. S3. Gene expression changes do not correlate with other methylation-related quantities such as total mCG and mCG mean. (A) Violin plots show that changes in gene expression in cell lines expressing different levels of MeCP2 are very small. (B) Number of genes versus mCG density in gene bodies. Genes have been binned as in Fig. 1C (bin width 0.1bp). (C) Log2FC of gene expression relative to appropriate controls (ctr – unmodified controls; SCR – scrambled control, OE ctr – overexpression control) for all seven levels of MeCP2, plotted against mCG density at promoters. Genes have been binned according to their promoter mCG density (bin size = 0.1bp), with each point representing a mean Log2FC of all genes falling in that particular bin. Black line shows the maximum slope. The slope of Log2FC for promoter mCG shows minimal dependence on the level of MeCP2. (D) As in C but for total mCG. The slope of Log2FC does not show a clear dependence on the level of MeCP2. (E) As in C but for mCG mean. In all panels, error bars represent +/- SEM.

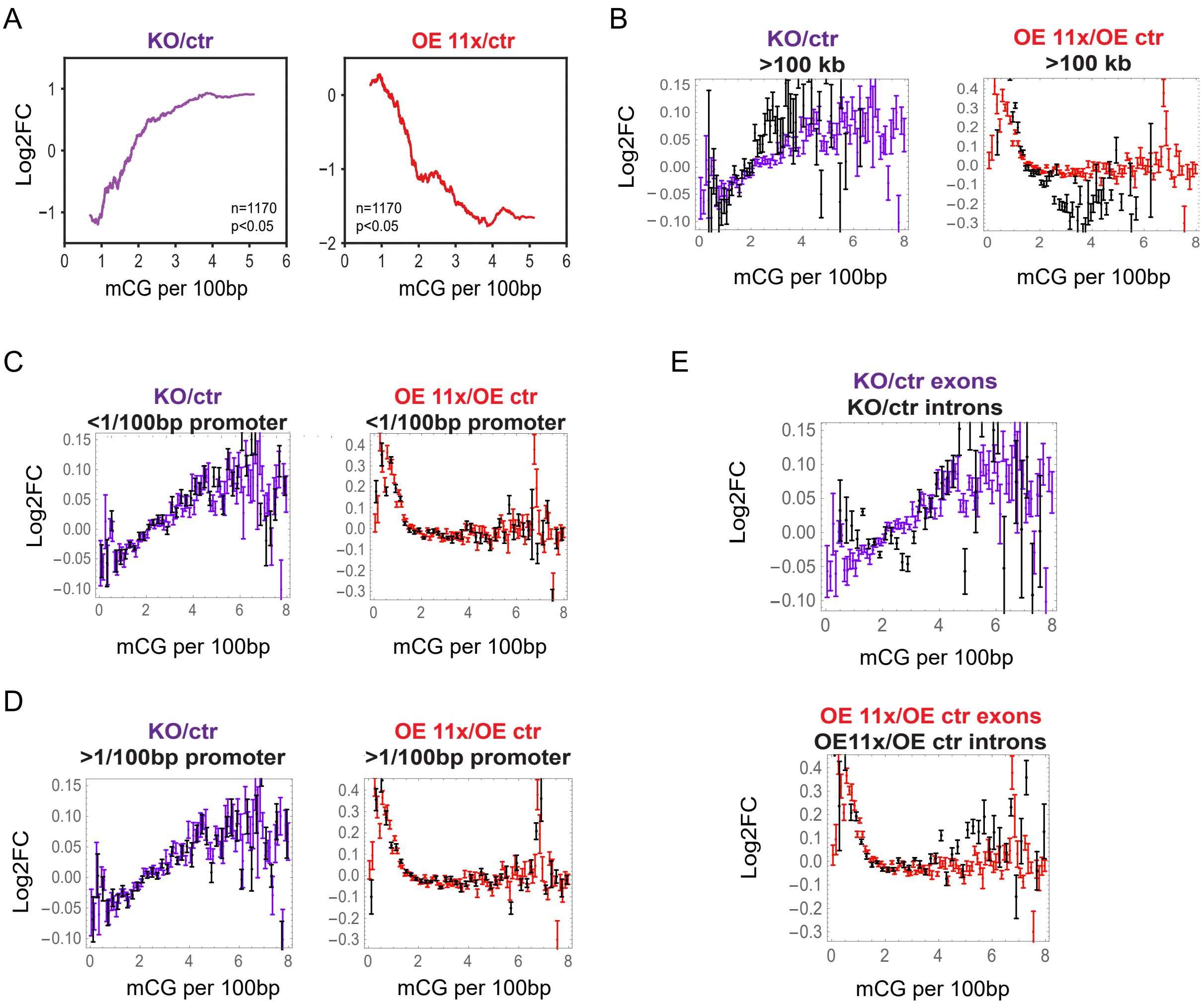

Fig. S4. Relationship of MeCP2 level with expression changes is robust. (A) Strong reciprocal correlation of Log2FC relative to appropriate controls for KO (purple, left panel) and OE (red, right panel) cell lines, plotted against gene body mCG density for significantly changing genes ($p<0.05$; 1170 genes). (B) Log2FC versus mCG density for KO/WT (left) and OE 11x/OE ctr (right) for genes longer than 100 kb (black) and all genes (purple and red) from Fig. 1C. (C) Log2FC versus mCG density for KO/WT (left) and OE 11x/OE ctr (right) for low promoter mCG density (<1/100bp, 6849 genes) (black); all data (purple and red). (D) As in (C) for high promoter mCG density (>1/100bp, 8528 genes). (E) Log2FC versus mCG density for KO/WT (top) and OE 11x/OE ctr (bottom) from intronic RNA (black), compared to exonic RNA (purple and red).

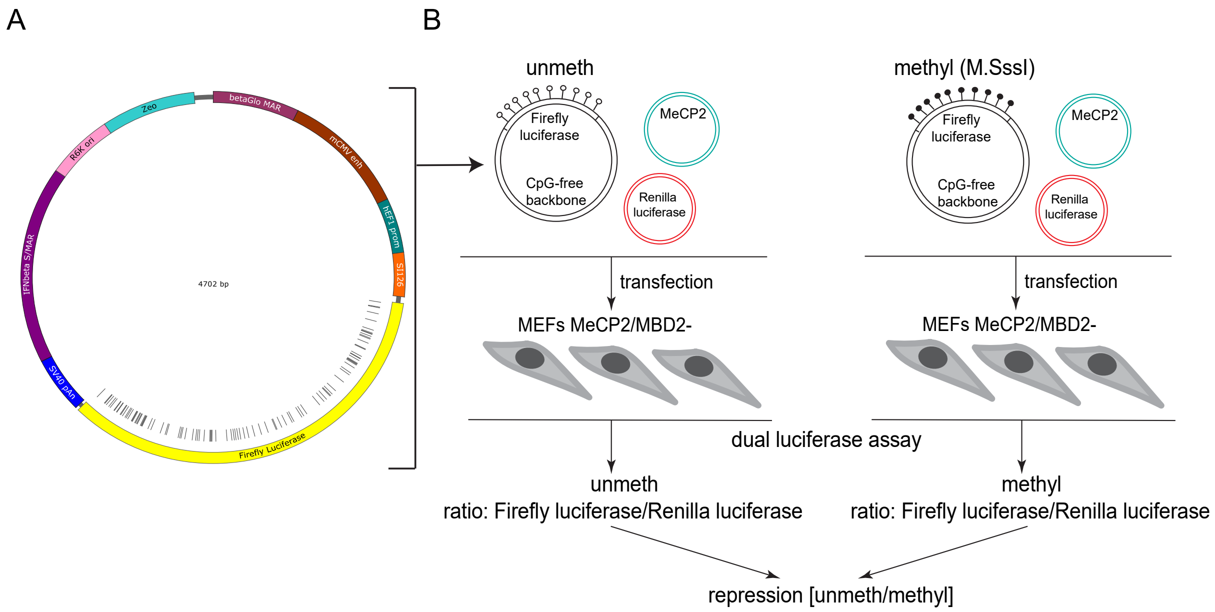

Fig. S5. Repression assay designed to measure MeCP2-mediated repression. (A) A map of the CpG-free vector and promoter used to express luciferase containing approximately 100 CpGs that are restricted to the body of the luciferase gene. (B) Study design shows how the expression of un-methylated and methylated reporter are compared, each normalised to a co-transfected construct expressing Renilla luciferase. Transfected mouse fibroblasts cells were null for both *Mecp2* and *Mbd2* genes.

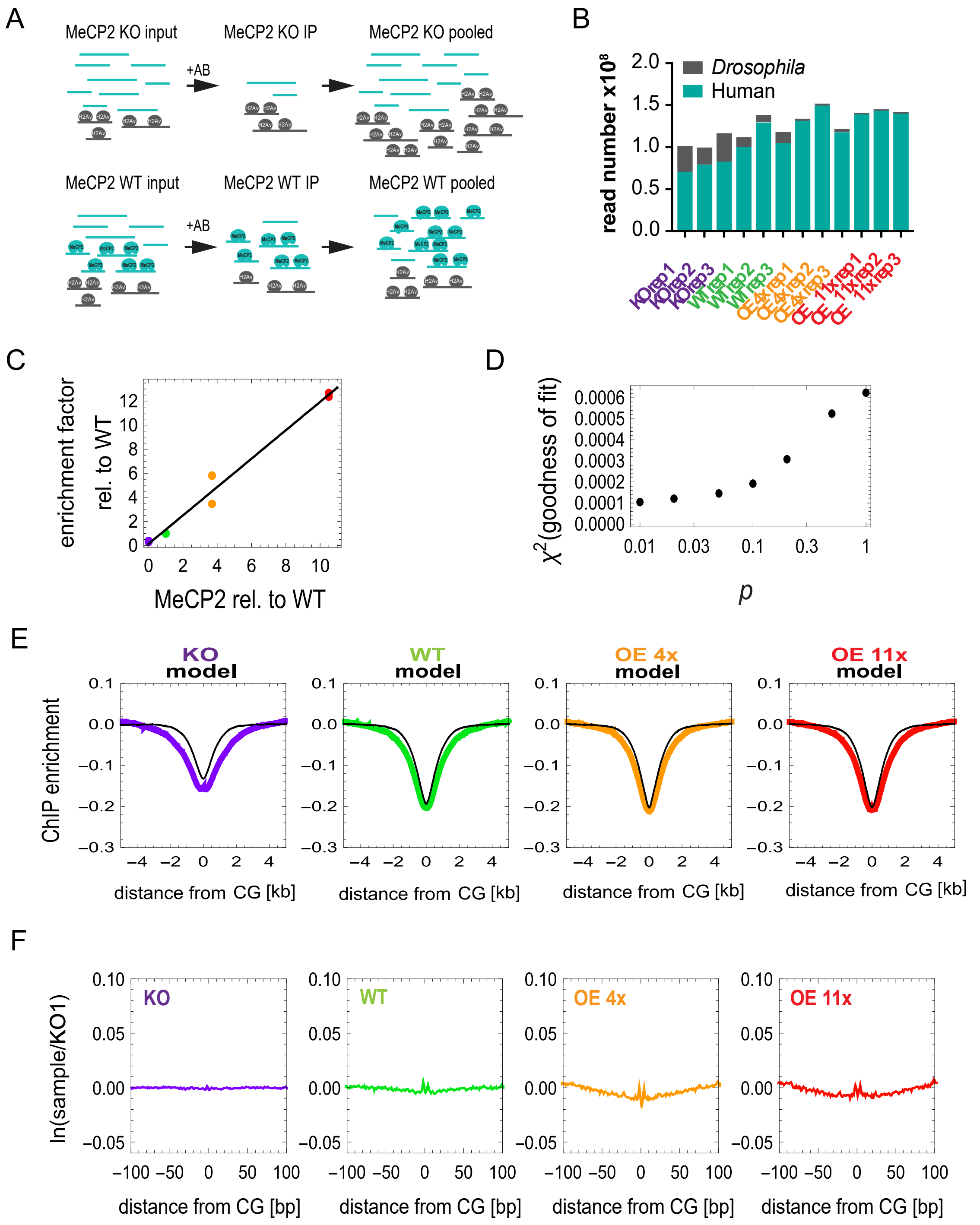

Fig. S6. Details of the experimental and simulated ChIP-seq and ATAC-seq. (A) Schematic representation of our quantitative MeCP2 ChIP-seq protocol for human neuronal chromatin (turquoise), with Drosophila chromatin spike-ins (grey) added for normalisation, and precipitated using antibodies against H2Av. (B) Total number of *Drosophila* reads compared to LUHMES reads obtained from ChIP-seq for cells expressing four levels of MeCP2: KO (purple), WT (green), OE 4x (orange) and OE 11x (red); each with three biological replicates. (C) Enrichment factor (human chromatin relative to spiked-in *Drosophila* chromatin) increases

linearly with the level of MeCP2. Two biological replicates are shown as individual data points. (D) ChIP-seq model goodness-of-fit versus p (probability that an mCG is occupied by MeCP2). The model becomes relatively insensitive to the exact value of p for $p < 0.1$ (similar values of χ^2). (E) ChIP-seq enrichment profiles centred at unmethylated CG dinucleotides. Black lines show profiles predicted by the model. (F) ATAC-seq depletion profiles in the +/-100 bp regions surrounding unmethylated CG dinucleotides. Profiles are averages over 2-4 biological replicates.

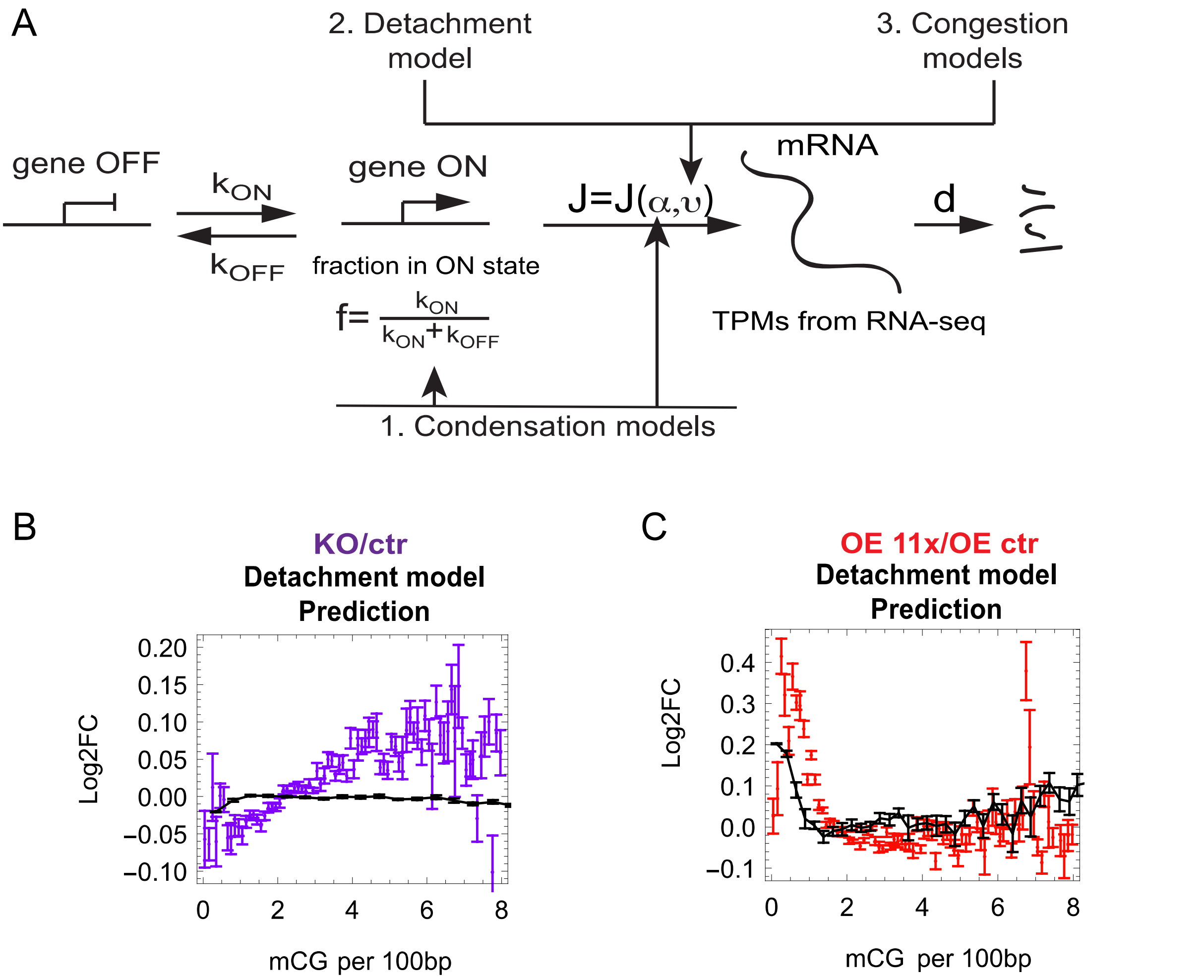

Fig. S7. Condensation and Detachment models fail to reproduce experimental data. (A) A general model of gene transcription. Each gene can be in two states: ON (TSS accessible to Pol II) or OFF (TSS not accessible). In the ON state, mRNA is produced with rate J which depends on the transcription initiation rate α and the elongation rate v. RNA-seq does not measure J but the amount of mRNA accumulated in the cell (TPM, transcripts per million) which also depends on degradation rate d. Three proposed models of MeCP2-dependent transcriptional regulation relate to different stages of transcription. (B) Log2FC predicted by the Detachment model (black line) does not agree with Log2FC for KO/ctr from RNA-seq (purple) when plotted against gene body mCG density. (C) Same as in (B) for OE 11x/OE ctr (red).

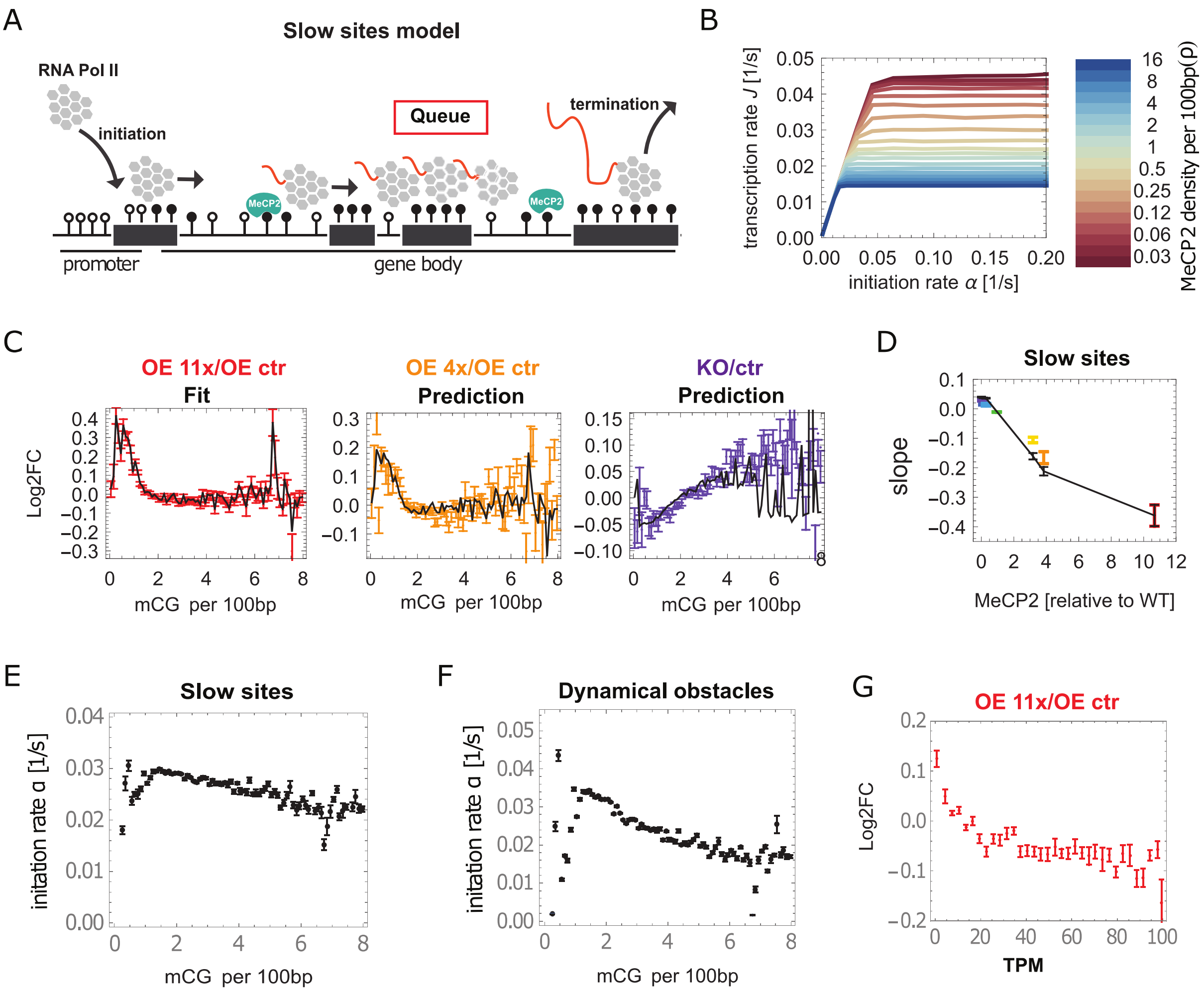

Fig. S8. Details of Congestion models. (A) The Slow Sites model in which MeCP2-induced chromatin modifications slow down elongating RNA Pol II. (B) Transcription rate J as a function of the initiation rate α. J saturates for large α, similarly as in the Dynamical Obstacles model (Fig. 4B). (C) Log2FC obtained from the Slow Sites model (black line) agree well with experimental data (red, orange and purple). Left panel: model fitted to OE 11x to obtain $\alpha(\rho)$. Middle and right panels: model predictions compared to the experimental Log2FC for OE 4x (orange) and KO (purple). (D) The maximum slope of Log2FC versus mCG density in gene bodies as predicted by the Slow Sites model (black line) reproduces the slopes from RNA-seq data for all seven levels of MeCP2. (E) Initiation rates obtained by fitting the slow sites model to Log2FC(OE 11x). (F) Initiation rates obtained by fitting the Dynamic Obstacles model to Log2FC(OE 11x). (G) Log2FC in OE 11x versus OE control plotted as a function of TPM in OE control shows a negative correlation with expression level as expected from the Congestion models.

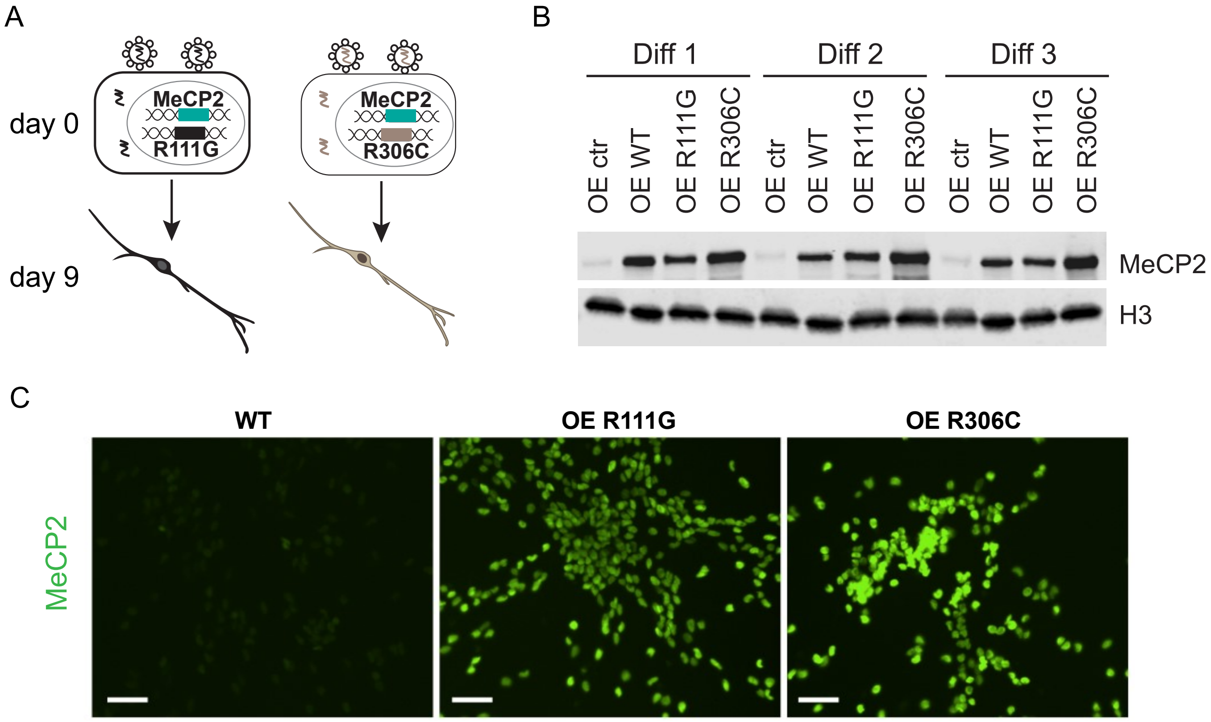

Fig. S9. Generation of MeCP2 overexpression mutants. (A) LUHMES cell lines were modified to overexpress MeCP2 with different mutations R111G (black) and R306C (brown). (B) Overexpression of R111G and R306C compared with control cells (OE ctr) and OE WT (11x) confirmed by Western blots using antibodies against MeCP2 and H3 as loading control in three independent differentiations. (C) Immunofluorescence images with an antibody against MeCP2 show uniform overexpression of MeCP2 mutants R111G and R306C in the LUHMES-derived neurons at 9 days of differentiation. Scale bar is 50 μm.

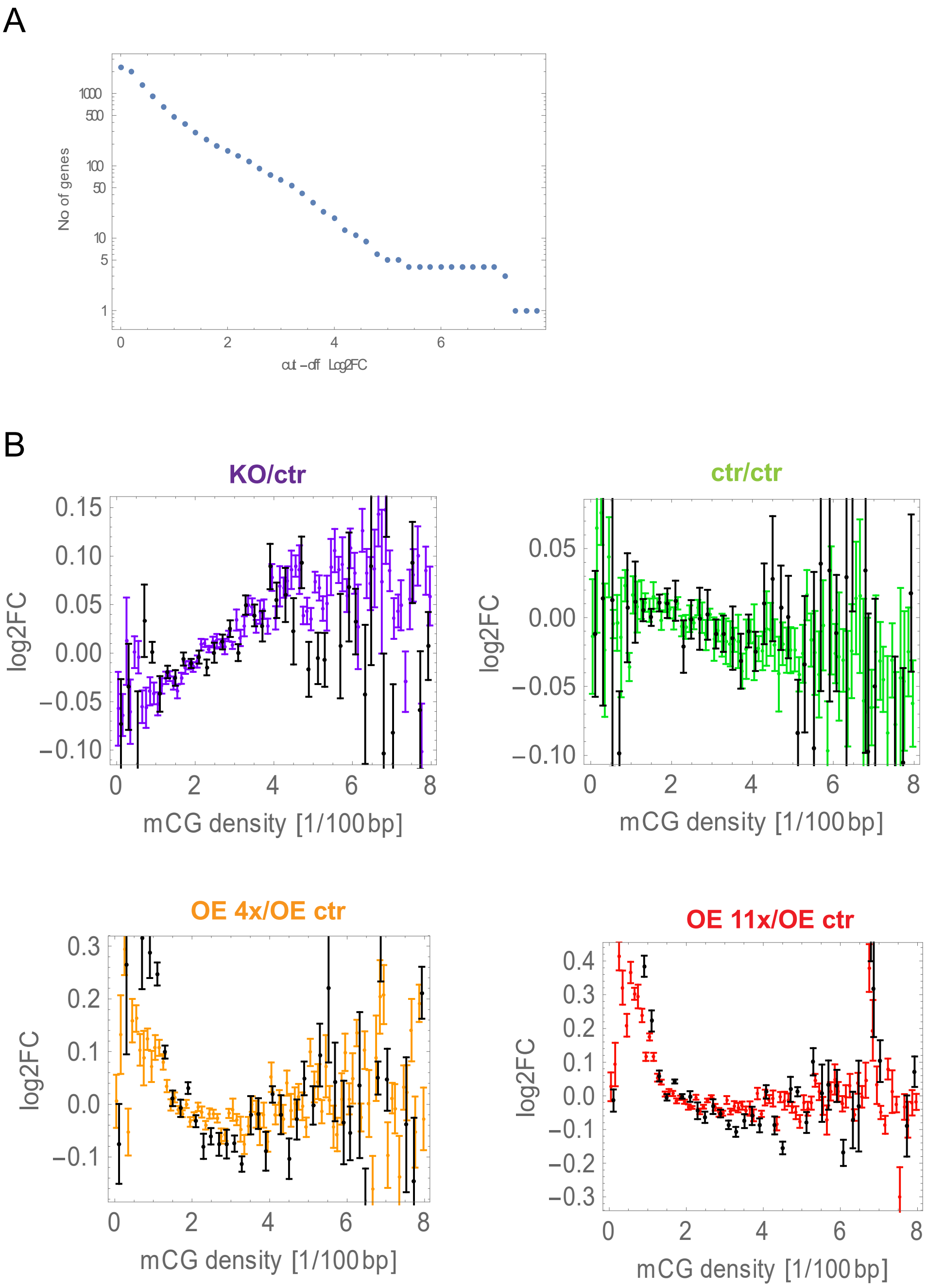

Fig. S10. (A) Number of genes for which Log2FCs are correlated with the level of MeCP2 (Spearman's r>0.8) and whose absolute value of Log2FC is larger than the cut-off Log2FC (horizontal axis). (B) Log2FC for genes changing monotonously (either increasing or decreasing) with MeCP2 level (black points). Unfiltered data from Fig. 1C is shown in colour.

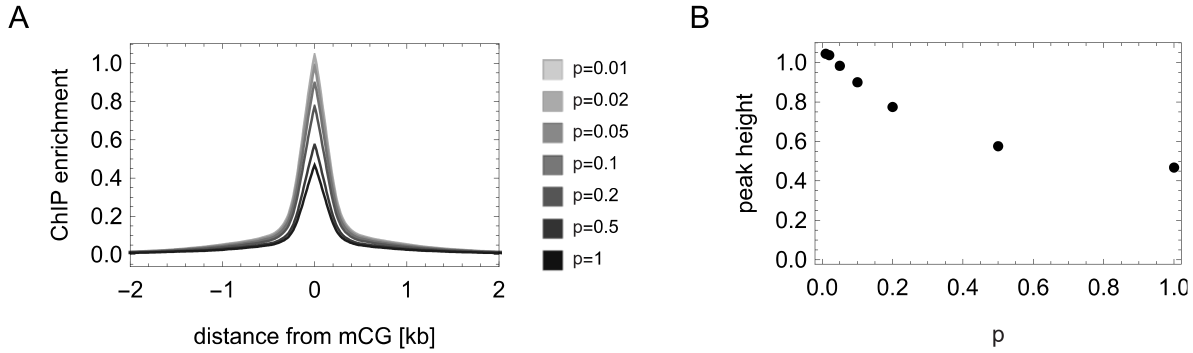

Fig. S11. (A) Simulated ChIP enrichment profiles for different MeCP2-DNA attachment probabilities p show a counter-intuitive reciprocal dependence between profile height and MeCP2 occupancy. (B) The height of the peak in the enrichment profile (C) as a function of p.

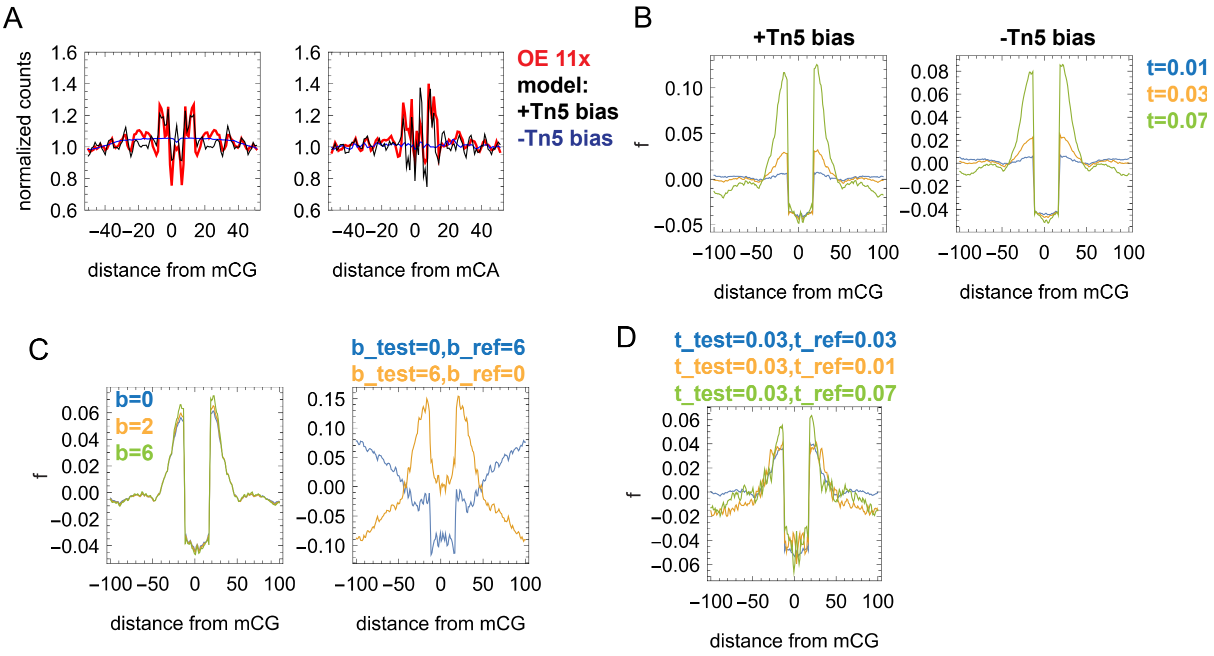

Fig. S12. Benchmarking of the simulated ATAC-seq. (A) Comparison between experimental and simulated ($p = 0, t = 0.04, g = 6$) ATAC-seq counts shows that Tn5 bias is important in reproducing the normalized counts *in silico*. Red = OE 11x, blue = simulated with no Tn5 bias, black = simulated with Tn5 bias. (B) Simulated footprint f for $p = 0.05, g = 6$ and $t = 0.01, 0.03, 0.07$ (blue, yellow, and green respectively) shows that excluding the Tn5 insertion bias does not significantly affect the depth of the footprint. Left = with Tn5 bias, right = no bias. (C) The role of CG bias b on the simulated footprint f. CG bias cancels out if identical in both test and reference samples. Left: the same $b = 0,2,6$ (blue, yellow, and green respectively) for the test and reference samples. The footprint is not affected by the bias. Right: different b's for the test and reference samples: $b_{test} = 0, b_{ref} = 6$ (blue) and $b_{test} = 6, b_{ref} = 0$ (yellow). The shape and depth of the footprint is significantly affected. In all cases $p = 0.04$. (D) The shape of the footprint depends on the difference in digestion time t between the test and the reference sample, but the depth of the footprint does not. Blue = ($t_{test} = 0.03, t_{ref} = 0.03$), yellow = ($t_{test} = 0.03, t_{ref} = 0.01$), green = ($t_{test} = 0.03, t_{ref} = 0.07$). In all cases, $p = 0.05, b = 6.0$.

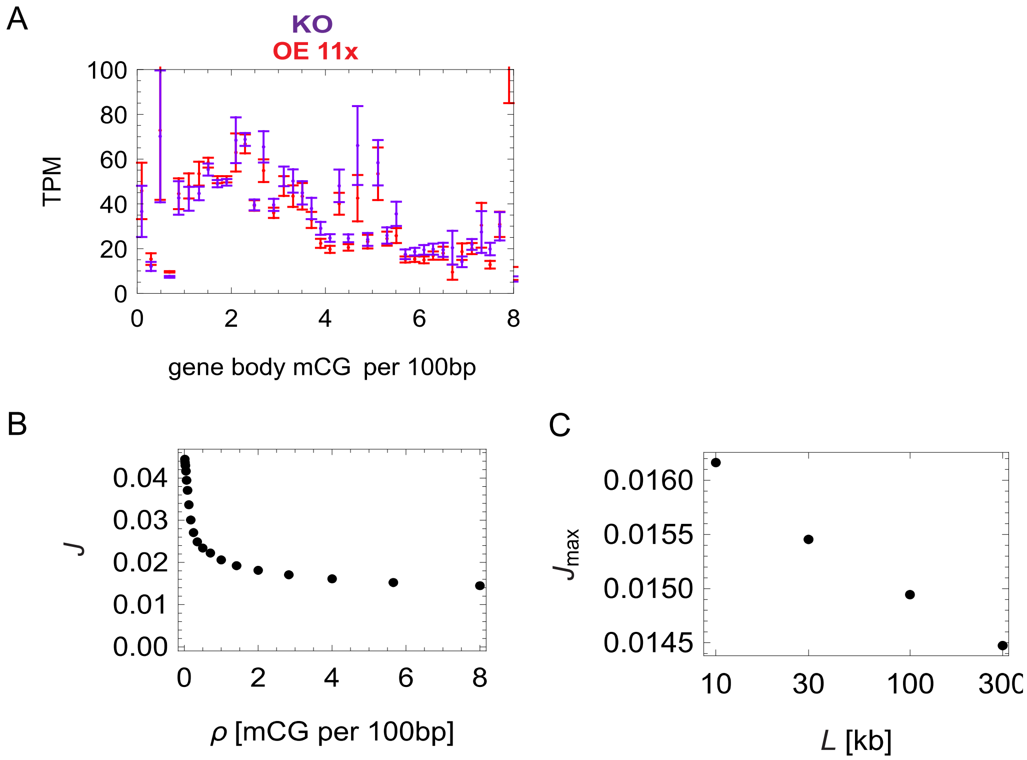

Fig. S13. (A) Plots of gene expression (transcripts per million, TPM) versus gene body mCG density for KO and OE 11x show that small differences between the two cell lines are overshadowed by a much stronger dependence on gene body mCG density that is independent of MeCP2. (B) Transcription rate J as a function of mCG density, for $L = 30$ kb. (C) Maximum transcription rate ($\rho = 0$) versus gene length L.

Table S1: Cell lines, their MeCP2 levels, and number of replicates for each experiment (RNA-seq, ATAC-seq, ChIP-seq). Note: replicate means independent differentiation.

			RNA-seq (number of replicates)					ATAC-seq (number of replicates)		ChIP-seq (number of replicates)	WGBS (number of replicates)		
cell line name	public name	MeCP2 relative to WT	Nov-13	Mar-14	Nov-14	Sep-15	Jan-17	Nov-15	Feb-16	Nov-16	BS_Jul_14	TAB_Sep_14	Ox_Mar_15
KO H4	KO1	0.06			4	4		1	1	2			
KO D10	KO2	0.05			4	4			2				
lenti 4 inf 1	KD1	0.11		4									
lenti 5 inf 1	KD2	0.33	3										
lenti 1 inf 1	Sc	0.87	3										
lenti 4 inf 2	KD1	0.13	3										
lenti 5 inf 2	KD2	0.24		4									
lenti 1 inf 2	Sc	0.90		4									
wildtype	WT	1.00	3	4					1	2	3	3	3
ctr E10	WT	0.95			4	4		1	1				
ctr F6	WT	0.79				4			2				
ctr C8	WT	0.82				4							
ctr G5	WT	1.06				4							
OEC CMV 32 A8	OEC	1.10				4	3						
lenti 24 inf 1	OE 3x	3.10	3										
lenti 24 inf 2	OE 3x	3.27		4									
OE Syn 35 E4	OE 4x	3.71				4			2	2			
OE Syn 35 E5	OE 4x	3.97				4							
OE CMV 33 E6	OE 11x	9.94				4		1	1				
OE CMV 33 C10	OE 11x	11.40				4	3			2			
OE CMV R111G 50 B8	OE R111G	7.21					3						
OE CMV R306C 49 C8	OE R306C	10.74					3						

Table S2: Sequences of shRNAs and guide RNAs used to make knock-downs and knock-outs.

name	sequence 5'-3'
shRNA1_s	ccggTGACAAAGCTTCCCGATTAACCTCGAGGTTAATCGGGAAGCTTTGTCAtttttG
shRNA1_as	aattCaaaaaTGACAAAGCTTCCCGATTAACCTCGAGGTTAATCGGGAAGCTTTGTCA
shRNA2_s	ccggACACATCCCTGGACCCTAATGctcgagCATTAGGGTCCAGGGATGTGTtttttG
shRNA2_as	aattCaaaaaACACATCCCTGGACCCTAATGCTCGAGCATTAGGGTCCAGGGATGTGT
shRNA_scr_s	ccggGCTAGAGAGTAATCCGTAGTAttcaagagaTACTACGGATTACTCTCTAGCttttttG
shRNA_scr_as	aattCaaaaaGCTAGAGAGTAATCCGTAGTAtctcttgaaTACTACGGATTACTCTCTAGC
Guide RNAs	
sgRNA A	AGAAGCTTCCGGCACAGCCG
sgRNA B	CGCTCCATCATCCGTGACCG

Table S3: The list of antibodies used in this work.

antibody	species	clonal	company	cat. number	method	concentration	RRID
anti-NF	mouse	mono	Covance	SMI-311R	IF	1:500 for IF	AB_509991
anti-H3	rabbit	mono	Cell Signaling	4499	WB	1:10 kx for WB	AB_10544537
anti-H3	rabbit	poly	Abcam	AB1791	WB	1:50 kx for WB	AB_302613
anti-MeCP2	mouse	mono	Active Motif	61286	IF	1:200 for IF	AB_2615067
anti-MeCP2	rabbit	mono	Cell Signaling	D4F3	WB/IF/ChIP	1000/200/50	AB_2143849
anti-MeCP2	mouse	mono	Sigma	6818	WB	1:1000 for WB	AB_262075
anti-MeCP2	mouse	mono	Sigma	7443	WB	1:1000 for WB	AB_477235

Table S4: q-PCR primers used in this work.

name	sequence 5'-3'
hMeCP2_1_f	gatcaatccccagggaaaag
hMeCP2_1_r	cctctcccagttaccgtgaa
hMeCP2_2_f	gagaccgtactccccatcaa
hMeCP2_2_r	agtcctttcccgctcttctc
hMeCP2_3_f	caaggccaaacagagaggag
hMeCP2_3_r	caatccgctccgtgtaaagt
hGAPDH_2_f	acccagaagactgtggatgg
hGAPDH_2_r	ttctagacggcaggtcaggt
hGAPDH_3_f	cagcctcaagatcatcagca
hGAPDH_3_r	tgtggtcatgagtccttcca
hCypA_1_f	ggtttatgtgtcagggtggtg
hCypA_1_r	ttctccccatagatggacttg
hCypA_2_f	ttttcatctgcactgccaag
hCypA_2_r	catggcctccacaatattca
hSox2_f	caagatgcacaactcggaga
hSox2_r	gcttagcctcgtcgatgaac
hFox-3_f	ccgaccctacagagaagcag
hFox-3_r	gaattgcccgaacatttgc
hTH_f	gtgttccagtgcacccagta
hTH_r	gccaatgtcctgcgagaa
hDAT_f	agtggcctggttctatggtg
hDAT_r	gaccacgaacaggagaaagc
hDRD2_f	ggaggtggtaggtgagtgga
hDRD2_r	gatgctgatggcacacaagt

Movie S1: Congestion model with dynamic obstacles: visualisation of Pol II traffic in the absence of obstacles.

Movie S2: Congestion model with dynamic obstacles: visualisation of Pol II traffic in the presence of MeCP2-induced obstacles (blue rectangles).

Movie S3: Congestion model with slow sites: visualisation of Pol II traffic in the presence of MeCP2-induced slow sites.

References

1. Bolger AM, Lohse M, Usadel B (2014) Trimmomatic: a flexible trimmer for Illumina sequence data. *Bioinformatics* 30(15):2114–2120.

2. Krueger F, Andrews SR (2011) Bismark: a flexible aligner and methylation caller for Bisulfite-Seq applications. - PubMed - NCBI. *Bioinformatics* 27(11):1571–1572.

3. Li H (2013) Aligning sequence reads, clone sequences and assembly contigs with BWA-MEM.

4. Ramírez F, et al. (2016) deepTools2: a next generation web server for deep-sequencing data analysis. *Nucleic Acids Research* 44(W1):W160–W165.

5. Dobin A, et al. (2013) STAR: ultrafast universal RNA-seq aligner. *Bioinformatics* 29(1):15–21.

6. Patro R, Mount SM, Kingsford C (2014) Sailfish enables alignment-free isoform quantification from RNA-seq reads using lightweight algorithms. *Nat Biotechnol* 32(5):462–464.

7. Liao Y, Smyth GK, Shi W (2014) featureCounts: an efficient general purpose program for assigning sequence reads to genomic features. - PubMed - NCBI. *Bioinformatics* 30(7):923–930.

8. Love MI, Huber W, Anders S (2014) Moderated estimation of fold change and dispersion for RNA-seq data with DESeq2. - PubMed - NCBI. *Genome Biol* 15(12):31.

9. Buenrostro JD, Giresi PG, Zaba LC, Chang HY, Greenleaf WJ (2013) Transposition of native chromatin for fast and sensitive epigenomic profiling of open chromatin, DNA-binding proteins and nucleosome position. *Nat Meth* 10(12):1213–1218.

10. Schep AN, et al. (2015) Structured nucleosome fingerprints enable high-resolution mapping of chromatin architecture within regulatory regions. *Genome Res* 25(11):gr.192294.115–1770.

11. Rabani M, et al. (2011) Metabolic labeling of RNA uncovers principles of RNA production and degradation dynamics in mammalian cells. *Nat Biotechnol* 29(5):436–442.

12. Blythe RA, Evans MR (2007) Nonequilibrium steady states of matrix-product form: a solver's guide. *J Phys A: Math Theor* 40(46):R333–R441.

13. Derrida B (1998) An exactly soluble non-equilibrium system: The asymmetric simple exclusion process. *Physics Reports* 301(1-3):65–83.

14. Krug J (1991) Boundary-induced phase transitions in driven diffusive systems. *Phys Rev Lett* 67(14):1882–1885.

15. Fuchs G, et al. (2014) 4sUDRB-seq: measuring genomewide transcriptional elongation rates and initiation frequencies within cells. *Genome Biol* 15(5):R69.
